# Heavy Atom Tunneling in Organic Reactions at Coupled Cluster Potential Accuracy with a Parallel Implementation of Anharmonic Constant Calculations and Semiclassical Transition State Theory

**DOI:** 10.1021/acs.jctc.1c01143

**Published:** 2022-01-07

**Authors:** Giacomo Mandelli, Chiara Aieta, Michele Ceotto

**Affiliations:** Dipartimento di Chimica, Università degli Studi di Milano, via C. Golgi 19, 20133 Milano, Italy

## Abstract

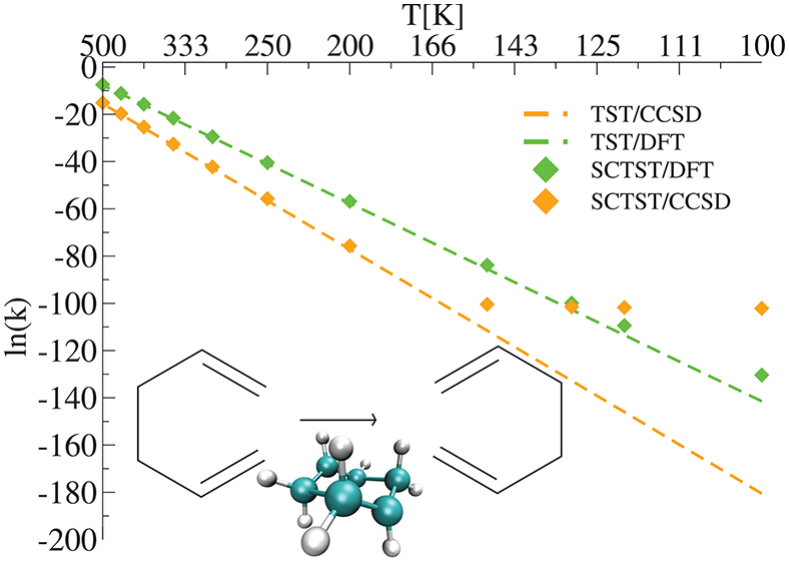

We describe and test on some organic reactions a parallel implementation strategy to compute anharmonic constants, which are employed in semiclassical transition state theory reaction rate calculations. Our software can interface with any quantum chemistry code capable of a single point energy estimate, and it is suitable for both minimum and transition state geometry calculations. After testing the accuracy and comparing the efficiency of our implementation against other software, we use it to estimate the semiclassical transition state theory (SCTST) rate constant of three reactions of increasing dimensionality, known as examples of heavy atom tunneling. We show how our method is improved in efficiency with respect to other existing implementations. In conclusion, our approach allows SCTST rates and heavy atom tunneling at a high level of electronic structure theory (up to CCSD(T)) to be evaluated. This work shows how crucial the possibility to perform high level ab initio rate evaluations can be.

## 1 Introduction

Calculation of reaction rates in theoretical chemistry is still nowadays a challenging task. The rate is an intrinsically dynamical quantity, and rigorous methods to compute reaction rates need to rely on dynamics simulations.^1^ However, this approach is complicated by the low probability of reactive events occurring in a typical time span of dynamics simulations.^2,3^ Moreover, quantum effects, such as tunneling and zero point energy, are recognized to have a significant impact on the rate constant value, especially at low temperatures. Therefore, in the cases where quantum effects are important, the quantum mechanical evolution of the system is mandatory.^4^ Obviously, this is a very complicated task,^5−12^ and it is not feasible to apply exact quantum methods for practical purposes, for instance, in kinetic modeling applications of complex systems.

Transition state theory (TST) is a clever rate constant approximation that avoids dynamics simulations and delivers rates in terms of static thermodynamics information.^13,14^ Since TST is a classical mechanics theory, early theories based on one-dimensional potential approximation were elaborated to account for quantum tunneling, such as Wigner or Eckart corrections.^15,16^ Nowadays, more sophisticated approximations have been developed to include, at least to some extent, the effects neglected by 1D approaches to tunneling corrections and limitations of the TST method itself, such as corner cutting, nonseparability of the reaction coordinate, and recrossing.^17−28^

Among these techniques, semiclassical transition state theory (SCTST) initially developed by W. H. Miller in the 1970s and revisited in the 1990s^29−32^ has received renewed attention in the past few years.^33−37^ What makes SCTST particularly convenient for application is that it requires input quantities that are routinely calculated by quantum chemistry codes. These include the harmonic vibrational frequencies, the height of the reaction barrier, and the anharmonic vibrational coupling constants, which are employed in the context of second order vibrational perturbation theory (VPT2). A user-friendly implementation of SCTST is provided along with the Multiwell program suite.^38−40^ Recently, the computational convenience of this program has been enhanced by a parallel implementation of the calculation of the vibrational density of states,^41,42^ which are used for the SCTST rate calculations. However, a main computational bottleneck still remains, and it is the calculation of the anharmonic couplings, which we will call below χ_*kk*′_. For these reasons approximate reduced dimensionality versions of the SCTST theory have been explored.^43−45^

In this work we propose instead to retain the full dimensional anharmonic couplings matrix to calculate the SCTST rates and develop a convenient parallel implementation to speed up their estimate. The possibility to parallelize this task has already been exploited for spectroscopic applications of VPT2, but it has been applied only to minimum geometries on the potential energy surface (PES).^46^ Our implementation extends the method for accelerated anharmonic constant calculations to transition state geometries. In addition, some more specific developments, such as the inclusion of Coriolis ro-vibrational couplings in the calculation of the total anharmonic constants matrix and a detailed treatment of the Fermi resonances along with the deperturbation process, are presented and implemented.

Eventually, our software allows us to compute SCTST rate constants of reactions of medium-high dimensionality at a high level of electronic structure theory with an affordable computational effort. We apply our implementation to the full-dimensional rate calculation of three heavy atom tunneling reactions, respectively composed of 10, 14, and 16 atoms, for which a fitted PES is not available. We carried out our electronic structure calculations at the level of density functional theory (DFT) and second order Møller–Plesset perturbation theory (MP2). For the 10-atom system, we were able to use coupled cluster with a full treatment of singles and doubles and an estimate to the connected triples contribution (CCSD(T)) level of electronic structure theory, while we used CCSD for the 14- and 16-atom systems.

The paper is organized as follows. In Section 2, we recall the SCTST theory and the expressions of the anharmonic couplings in the case of both a minimum structure and a transition state one. In Section 3, we describe in details our implementation, and we show the speedup performance of our parallel program. In Section 4, we describe some applications, and we compare our results with those obtained by other techniques. Eventually, in the Summary and Conclusions section (Section 5), we provide our final remarks and anticipate some future perspective.

## 2 Semiclassical Transition State Theory

The exact kinetic rate constant can be written as^47−49^
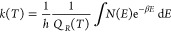
1where *N*(*E*) is the cumulative reaction probability (CRP), *h* is the Plank constant, *Q*_*R*_(*T*) is the reactants partition function, and β = 1/(*k_B_T*), where *T* and *k*_*B*_ are, respectively, the temperature and the Boltzmann constant. In the SCTST frame the CRP can be written using a multidimensional generalization of the one-dimensional WKB (Wenzel, Kramer, Brillouin) tunneling probability *P*(*E*) approximation:^50^
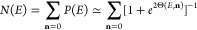
2where we consider a molecular transition state composed by *N*_*a*_ atoms with *N* = 3*N*_*a*_ – 6 vibrational degrees of freedom. In eq 2, ***n*** is a vector of *N* – 1 quantum integer numbers that defines the vibrational state of the transition state bound coordinates, and ∑_**n**=0_ = ∑_*n*_1_=0_ ∑_*n*_2_=0_... ∑_*n*_*N*–1_=0_ stands for the sum over all vibrational states. Θ(*E*, **n**) is the barrier penetration integral which needs to be determined to calculate *N*(*E*) and the rate constant. For nonseparable systems, the total energy *E* can always be expressed as
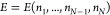
3and in the case of a transition state structure by means of the Bohr Sommerfeld quantization rule,^30^ the following identity holds for the *N*th reactive mode
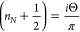
4This last relation allows to write the total energy as a function of the penetration integral *E*(*n*_1_, ..., *n*_*N*–1_, Θ), and by inversion it is in principle possible to get Θ(*E*, **n**).

Miller and Hernandez^31^ practically addressed this inversion problem by exploiting the standard perturbative expression^51^ for the vibrational energy levels in a minimum of the PES given by
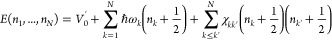
5where *V*_0_^′^ is the potential energy at the stationary point of the PES with the inclusion of a constant  term arising from the derivation of this expression in VPT2 context.^35,52,53^ ω_*k*_ are the normal-mode frequencies, and χ_*kk*′_ are the anharmonic constants. Using eq 4 and generalizing eq 5 to the case of a saddle point geometry, we can find an explicit form for the barrier penetration integral as a function of the total energy and the quantum numbers (*n*_1_, *n*_2_, ..., *n*_*N*–1_) related to the bound degrees of freedom:
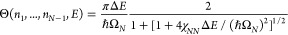
6where
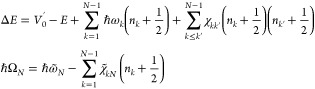
7For the *N*th imaginary mode we have that ω_*N*_ = *iω̃*_*N*_, χ_*kN*_ = −*iχ̃*_*kN*_, and .

Using eq 6, we evaluate the sum in eq 2 and get the CRP *N*(*E*). Then, by putting the calculated *N*(*E*) into eq 1, we finally get the rate constant.

In this work we compute the rate with the Multiwell program suite by separating the contribution from the different degrees of freedom to the partition functions.^38^ The SCTST rate constant is evaluated as
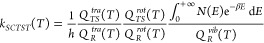
8where *Q*_*TS*(*R*)_^*tra*^(*T*) is the transition state (reactant) translational partition function and *Q*_*TS*(*R*)_^*rot*^(*T*) is the transition state (reactant) rotational one. These are approximated to the corresponding free motion partition functions,
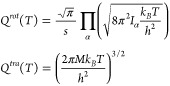
9where *s* is the rotational symmetry number, *I*_α_ is the moment of inertia along the α = *x*, *y*, *z* axis, and *M* is the total mass of the reactant or the transition state.

The vibrational partition function of the reactants is fully coupled and anharmonic since it is written in terms of the reactant density of vibrational states (DOS) ρ(*E*) as
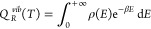
10For the numerator in eq 8, the calculation of the semiclassical *N*(*E*) is not trivial. A practical way to address this problem is to divide the energy range of interest into bins of width δ*E*.^33^ In this way a certain number #_*j*_ of energy levels, each one identified by a combination of quantum vibrational numbers ***n*** = ***ñ***, will be found in the *j*th bin, and the corresponding average reaction probability is defined as
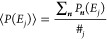
11Therefore, eq 2 is rewritten as
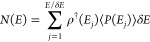
12where ρ^†^(*E*_*j*_) is the vibrational density of states (DOS) associated with the real-valued frequency vibrations of the TS. Note that, as δE is reduced, the result becomes more accurate. Within this approximation, the sum in eq 2 over the accessible states is replaced by the easier sum over *E*/δ*E* energy bins with energy lower than or equal to the total energy *E*. As a result, the rate constant calculation is reduced to the problem of evaluating two vibrational DOSs, ρ(*E*) for the reactants and ρ^†^(*E*_*j*_) for the TS. To achieve a convenient computational effort to estimate the SCTST rate, some of us have recently implemented a parallel version of the density of vibrational states (DOS).^41,42^ However, the main computational bottleneck in the entire approach still remains the evaluation of the anharmonic constants χ_*kk*′_.

We now survey how to derive an explicit and convenient form for the anharmonic constants that we will use in our implementation. Equation 5 is a Dunham expansion^54^ of the energy for a molecular system where vibrational and rotational motions are coupled. The corresponding rovibrational Hamiltonian, which includes the rotational kinetic energy, has the following form^55^
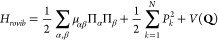
13where α and β are the rotational axis indices, μ is the inertia tensor, Π is the vibrational angular momentum operator, *k* is the normal mode index, and *V*(**Q**) is the potential in normal coordinates. **Q** is obtained as an orthogonal transformation of the mass weighted Cartesian displacement coordinates, and ***P*** is the conjugated momentum operator. The leading term of this Hamiltonian is the harmonic one and can be written in dimensionless normal coordinates as^52,53^
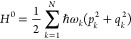
14with *q*_*k*_ = γ_*k*_^1/2^*Q*_*k*_ and , where *c* is the speed of light and *p*_*k*_ = (ℏω_*k*_)^−1/2^*P*_*k*_. Given this harmonic leading term, we are allowed to write the potential as a Taylor expansion in *N* normal coordinates at the equilibrium point *V*_0_. If we truncate this expansion at the 4th order, we get the following quartic force field (QFF) form of the potential
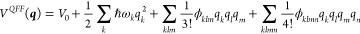
15where ϕ_*klm*_ and ϕ_*klmn*_ are the force constants. ϕ_*klm*_ is related to the third order potential derivatives as
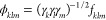
16where *f*_*klm*_ is the third order derivative along the three normal modes:
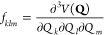
17

Analogous formulas hold for the potential fourth derivatives ϕ_*klmn*_.

Using the n-Mode coupling Representation (n-MR) notation recalled in Appendix A, we can rewrite eq 15 as
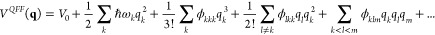
18where *V*_0_ is the potential energy at the stationary point of the PES.

The advantage of eq 18 is that it allows us to order the terms of the *V*^*QFF*^(***q***) expansion as a function of the number of modes coupled in the potential derivatives. Then, we can truncate the expansion depending on the desired number *n* of coupled terms in the potential to obtain the *n*-mode coupling representation of the quartic force field (nMR-QFF). In this way, the potential is better represented for a perturbation treatment, taking the harmonic terms as the zero-order leading ones. The anharmonic terms from the potential will cause the total Hamiltonian matrix to have nonzero off-diagonal terms. Finally, using the van Vleck perturbation theory (VVPT),^53^ we can formulate the vibrational perturbation theory of the second order plus resonances (VPT2+K) expression of the anharmonic constants reported in eq 5 at the transition state geometry
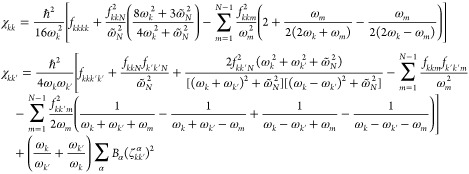
19with *k*, *k*′ = 1, 2, ..., *N* – 1 and χ_*kN*_ = −*iχ̃*_*kN*_. At the equilibrium geometry, the anharmonic constants are calculated as follows
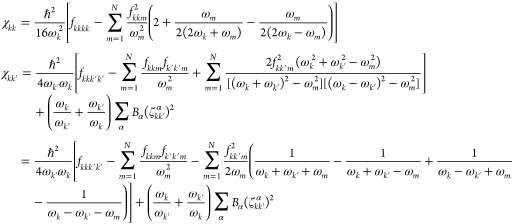
20where now *k*, *k*′ = 1, 2, ..., *N* since all frequencies are real. In eqs 19 and 20, *B*_α_ is the rotational constant with respect to the α rotational axis, and ζ_*kk*′_^α^ is the related Coriolis coupling tensor. To simplify the notation, in eqs 20 and 19 we use the same symbol ω for vibrational frequencies and χ anharmonic couplings for both equilibrium geometries and TS ones. In our code implementation, eqs 20 and 19 are factorized to remove possible resonances, as it will be explained in detail below. The VPT2+K formulation of the anharmonic constants requires a 3MR-QFF, and this is the potential approximation that we will use in the present paper.

## 3 Implementation

### 3.1 Our Software Workflow

**Figure 1:**
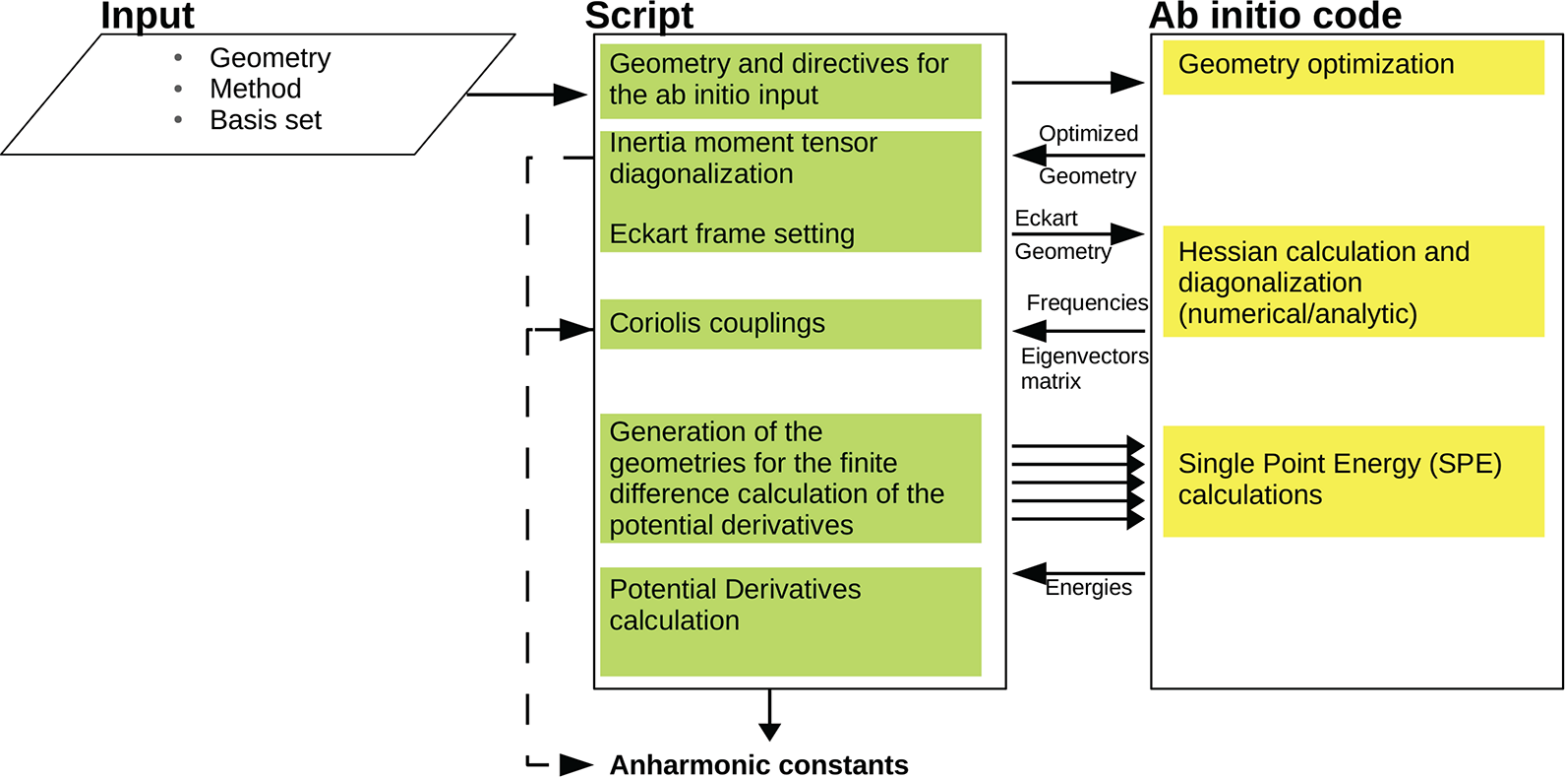
General workflow of the program. The two main communicating blocks are the script implemented in this work (green) and the ab initio program (yellow). Plain arrows between different blocks highlight the input–output flow between the script and the ab initio code. Dashed arrows track the information flow to compute Coriolis contributions to the anharmonic constants.

Our implementation consists in a script that interfaces with any ab initio quantum chemistry software. The script exploits a parallel architecture to compute the VPT2+K anharmonic constants for a transition state or a stable molecular geometry. The program workflow is shown in Figure 1, where the interface with the ab initio electronic structure code is detailed. Specifically, once the user has provided an initial geometry and the desired level of electronic structure theory (input box), our software automatically composes the appropriate input file for the ab initio code which performs the geometry optimization. The script sets strict optimization thresholds and appropriate grid densities in case of DFT calculations in order to have reliable anharmonic constants, as described in Supporting Information Section 1.^52^ Then, our script calculates the inertia moment tensor from the optimized geometry to set the molecule in the Eckart frame.^56^ This frame is characterized by two constraints. The first one implies that the origin of the system is placed at the system center of mass. The second condition enforces that the total angular momentum in this frame is zero. Our software first calculates the center of mass
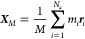
21where ***X***_*M*_ is the vector of the center of mass coordinates, *m*_*i*_ is the mass of the *i*th atom, and ***r***_*i*_ = (*r*_*i*,*x*_, *r*_*i*,*y*_, *r*_*i*,*z*_) is the position vector of the *i*th atom. Then, the coordinates of the molecule are centered in ***X***_*M*_, and the inertia tensor of the molecule is calculated according to Appendix B. The moment of inertia tensor is then diagonalized, and the orthonormal eigenvectors are found. In the last step, the coordinates of the molecule are transformed into the frame defined by these eigenvectors. Within the Eckart frame, the Hessian is calculated either by using the coupled ab initio code and its analytical gradients or by finite differences. The Hessian matrix is then diagonalized. We call the corresponding vibrational eigenvectors matrix **l** and the eigenvalue diagonal matrix ***W***.

In the next step, the Coriolis couplings are calculated. The Coriolis terms ζ_*kk*′_^α^ are matrix elements related to the interactions between the rotational and the vibrational motion. These are originated by the contribution arising from the rovibrational coupling as in the kinetic energy of the system^57,58^
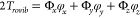
22where φ_*i*_ are the angular velocities of the rotating system of axes and
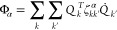
23where ***Q***^*T*^ = (*Q*_1_, *Q*_*k*_, ..., *Q*_*N*_)^*T*^ is the transpose vibrational normal coordinate vector and ***Q̇*** its time derivative vector. ζ_*kk*′_^α^ are the Coriolis coupling matrices
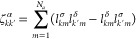
24where α, σ, and δ are the rotational axes, the indices *k* and *k*′ represent the *k*th and *k*′th normal mode, and *l*_*k*(*k*′)*m*_ is the *k*(*k*′)*m*-component of the Hessian eigenvector matrix **l** defined above. It is not necessary to include in eq 23 the translational and rotational contributions since the system of reference is the Eckart frame.

At this stage, the script is ready for the generation of the displaced geometries to compute by finite differences the third and fourth order derivatives of the potential. This last process is the parallel core of our implementation, and it is detailed in the next paragraph. Once the potential derivatives have been calculated, they are combined with the Coriolis coupling matrices to compute the anharmonic couplings according to eq 19 or 20.

### 3.2 Parallelization of the Derivative Calculations

**Figure 2:**
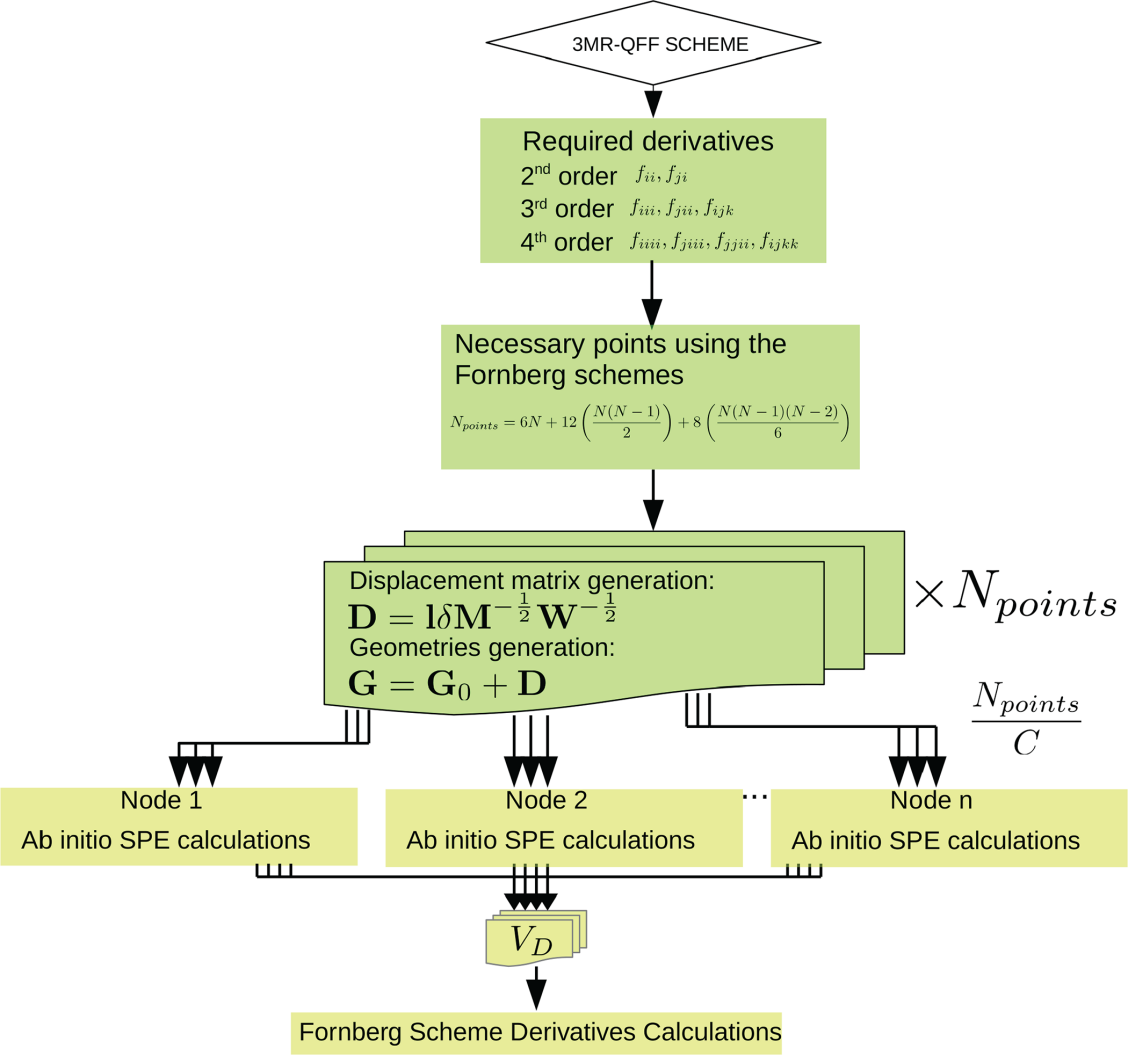
Algorithm implemented for the calculation of the third *f*_*ijk*_ and fourth *f*_*iijk*_ order derivatives of the potential. *N* is the number of vibrational degrees of freedom for the molecule, **D** is the displacement matrix, **l** is the columnwise vibrational eigenvector matrix, δ is a fixed incremental step, **M** is the diagonal matrix containing the atomic masses, and ***W*** is the diagonal vibrational eigenvalues matrix. **G** is the displaced geometry vector, and **G**_0_ is the equilibrium geometry vector. After the generation of the geometries, the SPE calculations are launched. *N*_points_ is the total number of energy points required, *C* is the number of cores available per node, and **V**_*D*_ is the vector that contains all the energies retrieved from the SPE calculations.

The specific parallel algorithm used to compute the potential derivatives is shown in Figure 2. The main idea is to perform parallel single point energy (SPE) calculations after generating a set of displaced geometries according to the finite difference scheme proposed by Yagi et al.^59^ for the computation of third and fourth order derivatives of the potential. Specifically, we derived the finite difference formulas from the Fornberg schemes.^60^ Mixed derivatives of the potential have been approximated up to the second order of accuracy, while the diagonal terms have been calculated at the fourth order of accuracy. This choice was originally proposed by Allouche et al.^46^, and it is convenient because it avoids any additional PES points for the fourth order approximation of the direct (not mixed) derivatives.

The Fornberg method for the generation of finite difference formulas on spaced grids is based on a simple recursion formula on 1-dimensional grids to determine the weights of the potential points in any derivative order formula and up to any order of accuracy. For our purpose we used an equally spaced grid along each normal mode. For multidimensional derivatives we combined the 1D formulas to get the corresponding multidimensional expressions. In this way, we obtained a finite difference expression for the derivatives *f*_*iii*_, *f*_*iiii*_, *f*_*iij*_, *f*_*iiij*_, *f*_*iijk*_, and *f*_*ijk*_ reported in Appendix C, which are necessary for anharmonic constants calculation according to eqs 19 and 20.

In a 3MR-QFF formula, the overall number of SPE calculations at different geometries is equal to
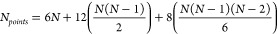
25To compute the coordinates of each configuration for the derivative calculations, the displacement matrix **D** is generated
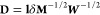
26where **M** is the diagonal matrix containing the atomic masses and δ is a fixed displacement. We fixed this displacement to 0.5 to get reliable results according to our tests and the literature.^59^ After identifying the equilibrium geometry with a 3*N*_*a*_ dimensional vector of Cartesian coordinates **G**_0_, each displaced geometry is generated as
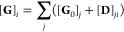
where **G**_*i*_ is the *i*th geometry vector component corresponding to the application of the *i*th displacement vector [**D**]_*ji*_ of eq 26 to the equilibrium geometry vector **G**_0_. Once all geometries necessary for the finite difference derivative calculation are generated, the SPE calculations are launched in parallel. The total number *N*_points_ of independent SPE ab initio inputs, automatically generated by the program, is divided into  launching files, where *C* is the number of computing cores available per node. The launching scripts are generated such that each node, composed by *C* physical cores, runs in a parallel fashion *C* SPE calculations. The energies are then retrieved and saved in a vector from which the third and fourth order derivatives of the potential will be computed using the schemes explained in Appendix C.

### 3.3 Resonance Treatment

The anharmonic constants are calculated using the GVPT2+K theory by implementing eqs 20 and 19. Specifically, in this step it is of fundamental importance to properly treat divergent terms that may arise from the zeroing of the denominators occurring at the resonances. The anharmonic constants can be affected by two different kinds of 1–2 resonances. In Fermi type I resonances between mode *i* and mode *j* we have
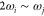
while in Fermi type II resonances an additional mode *k* is involved
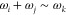
The usual way to deal with these resonances is to set a threshold and see if the difference in wavenumbers between the frequencies is smaller than this threshold. In this case, the resonant terms are set to zero in eqs 19 and 20. Unfortunately, this approach is not very accurate because it completely disregards the entire term, i.e., both numerator and denominator, even if it is only the denominator to be singular. Therefore, we decided to adopt the Martin et al.^61^ approach that is based on the evaluation of two parameters. For a Fermi type I resonance the parameter
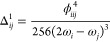
27and for a Fermi type II resonance
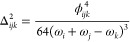
28If the Δ parameter is greater than a lower threshold (usually set at 1 cm^–1^) and the frequency difference between the modes involved in the resonance is smaller than a higher threshold (usually set at 200 cm^–1^), the resonant term is disregarded.

### 3.4 Parallel Implementation Scaling Benchmark

**Figure 3:**
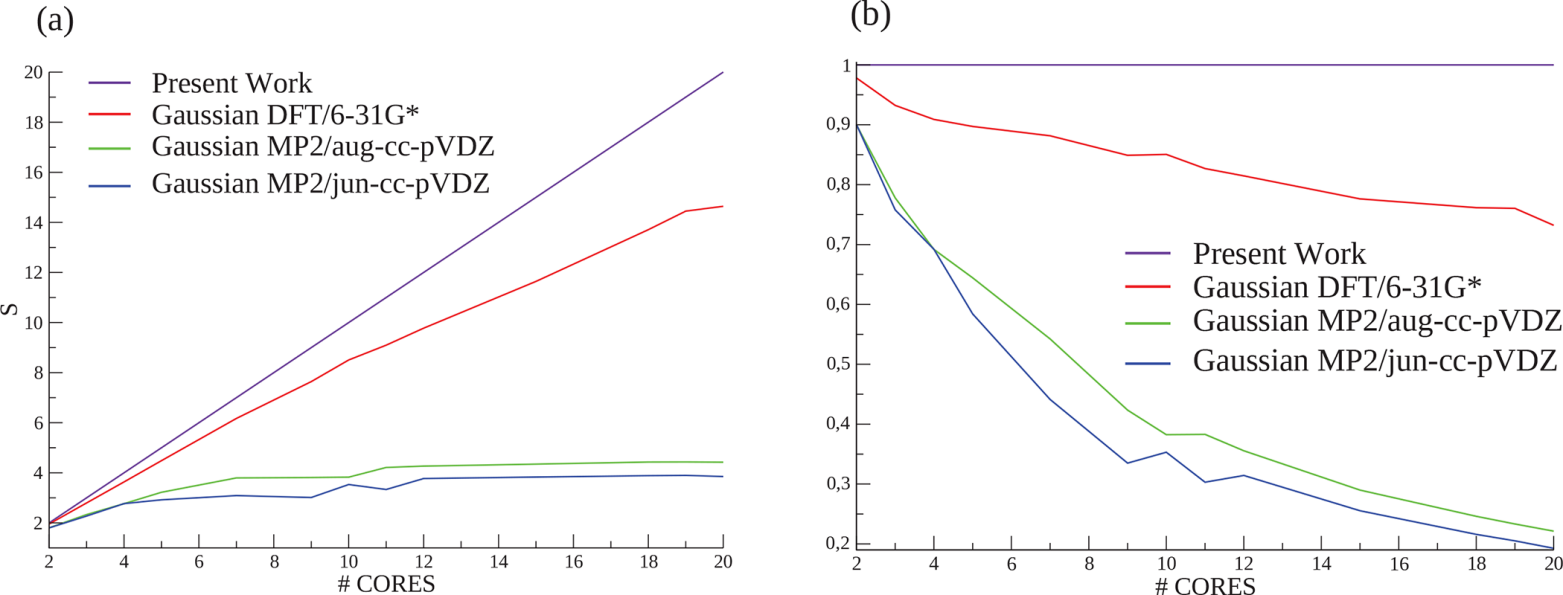
(a) Speedup and (b) efficiency plots for the anharmonic constants calculation using the Gaussian code and our program. Calculations are done for the 10 atom cyclobutene molecule at the MP2/aug-cc-pVDZ, MP2/jun-cc-pVDZ, and B3LYP/6-31G* levels of theory.

In this paragraph we will show how our program scales with respect to the number of processors. We compare the performance of our script interfaced with Gaussian 16 SPE calculations against Gaussian 16 internal routine for the anharmonic constant calculations.^62^ The scaling is rationalized in terms of speedup (*S*) and efficiency (*E*) parameters, which are respectively defined as
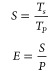
29where *T*_*s*_ is the serial execution time and *T*_*P*_ is the parallel one on *P* processors. The best speedup that one can get is linear with respect to the number of processors, and the efficiency is 1. However, this is hardly achieved in practice because of the core communication and disk writing/reading latency times present in parallel architectures. In our case the latency time is highly reduced, due to the parallelization strategy in which cores do not communicate with one another. Figure 3 reports our scalability tests for the 10-atom system, using either the MP2 or the DFT method, with aug-cc-pVDZ, jun-cc-pVDZ, and 6-31G* basis sets. Information about the specific architecture used for our benchmarks and further tests on the 14-atom system can be found in Supporting Information Section 2.

In Figure 3, the scaling *S* is linear for all kinds of computational setups when employing our algorithm. Indeed, the time spent by our script in the nonparallel part is negligible. The same calculation on the same architecture but using the Gaussian 16 software scales almost linearly only for a few cores. For a higher number of cores, the Gaussian 16 software parallel performance deviates form the ideal scaling. The same considerations are valid for the *E* profile, where the efficiency of the Gaussian 16 software drops with the increasing number of allocated CPUs. These considerations do not depend on the basis set employed. As far as the level of theory is concerned, DFT scales slightly better than MP2 when employing the Gaussian 16 software, especially for high dimensional molecules, but still far from linearity. For example, with the GALILEO IBM NeXtScale architecture by the Italian CINECA HPC center,^63^ the best performance for the anharmonicity constant computation of the *cis*-1,3,5-hexatriene at the MP2/aug-cc-pVDZ level of theory using the Gaussian 16 program was achieved over 12 cores, taking 32 h and 23 min. The same calculation on the same architecture was performed in 19 h with the best possible setup using our program. Furthermore, we point out that the calculation of the cyclobutene anharmonic constants at the CCSD(T)/aug-cc-pVDZ level of theory are not doable with Gaussian 16. Thus, we could not compare our code performance with Gaussian 16 in this case. For this CCSD(T) calculation the maximum number of parallel cores employed is actually limited by the RAM size available on each node. However, we experienced that a 252 core parallelization takes only 20 h to complete the calculation with our code.

Therefore, large molecule anharmonicity calculations are more conveniently addressed with our algorithm rather than with Gaussian 16, when a large amount of cores is available.

## 4 Results and Discussion

**Figure 4:**
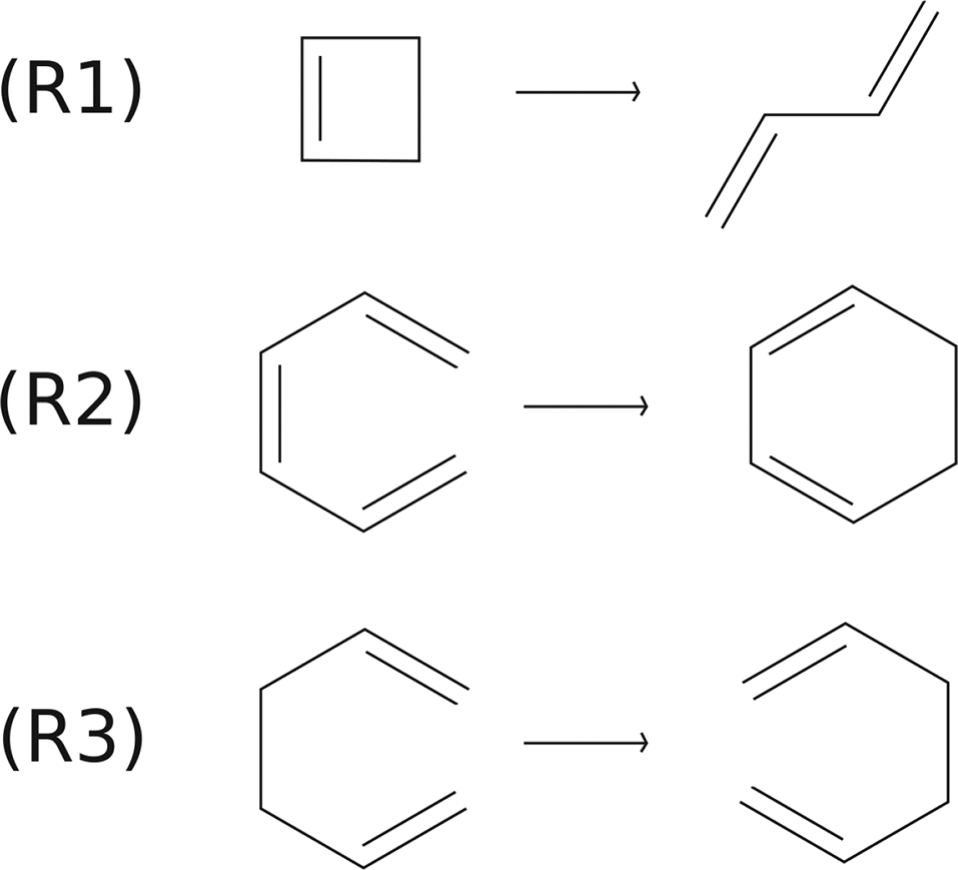
Organic reactions simulated in this work: The cyclobutene ring opening reaction (R1), the *cis*-1,3,5-hexatriene electrocyclic ring closing reaction (R2), and the [1,5]-Cope rearrangement of the 1,5-hexadiene molecule (R3).

In this section, we will calculate the rate constants using the anharmonicity matrices computed with our algorithm as input for the Parsctst and Paradensum codes^41,42^ of the Multiwell program suite.^38^ We decided to apply our algorithm to the study of the kinetic rate constants for the three organic reactions shown in Figure 4. The reactions involve 10, 14, and 16 atoms. They are the cyclobutene ring opening reaction (R1), the *cis*-1,3,5-hexatriene electrocyclic ring closing reaction (R2), and the [1,5]-Cope rearrangement of the 1,5-hexadiene molecule (R3). These reactions are known as examples of heavy atom tunneling (HAT) processes. HAT includes all the tunneling contributions to the reaction mechanism involving atoms that do not belong to the first period of the periodic table. These kinds of reactions have attracted a growing interest in recent years, both from the theoretical and the experimental community.^64−67^ Specifically, a recent paper by Greer et al.^68^ reports theoretical calculations of the tunneling contribution to the rate constant for these three reactions.

We start by validating our method. We compare the anharmonic constants calculated with our algorithm with those obtained using the internal Gaussian 16 subroutine. The aim is to check if possible differences may significantly impact the SCTST rate values. We define the difference and the percentage difference between the anharmonic constants obtained with our algorithm χ_*kk*′_^*prog*^ and the ones with the Guassian16 software χ_*kk*′_^*gau*^ as
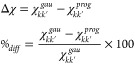
30where *k* and *k*′ are the normal mode indices.

**Figure 5:**
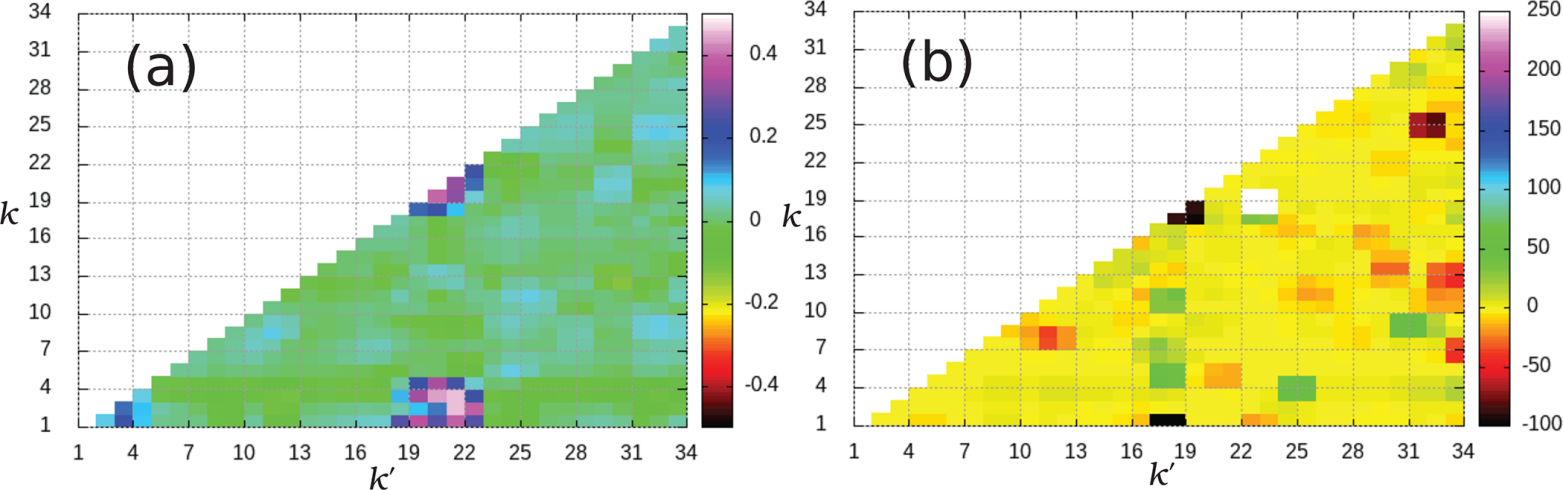
(a) Difference Δ*χ* between anharmonic constants calculated with Gaussian 16 and our program for the hexatriene molecule. (b) Percentage difference %_*diff*_ between the anharmonic constants. In both panels, *k* and *k*′ normal mode indices are reported on the axes, and the color gradient indicates the magnitude of the difference. Anharmonic constants are calculated at the MP2/aug-cc-pVDZ level of ab initio theory.

**Figure 6:**
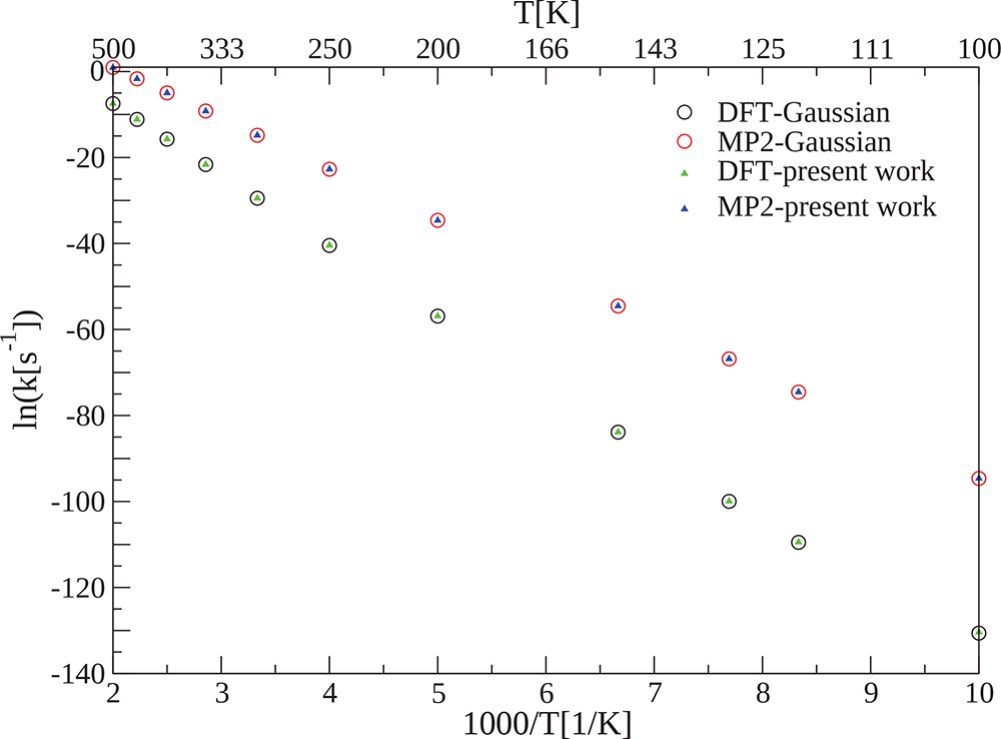
SCTST reaction rate constants *k*(*T*) at different temperatures for the [1,5]-Cope rearrangement (R3). The *y* axis reports the natural logarithm of the rate constants and the abscissa the scaled inverse temperature in Kelvin. The anharmonic constants matrix was calculated using both our program and the internal Gaussian 16 subroutine starting from the same geometry and ab initio calculation setup. DFT calculations have been carried out using the B3LYP/6-31G* setup, while MP2 calculations use the aug-cc-pVDZ basis set.

Figure 5 presents our results at the MP2/aug-cc-pVDZ level of theory. The axes in Figure 5 refer to the indices *k* and *k*′ of the normal modes, and the color gradient indicates the magnitude according to the label. Other B3LYP/6-31G* and MP2/aug-cc-pVDZ calculations have been carried out for the R1, R2, and R3 reactants and TS geometries, as reported in Supporting Information Section 3, and they show comparable deviations. Few values show more than 100% deviation from the Gaussian 16 ones. These values are all related to small anharmonic couplings. Anyhow, when the rate constant is calculated, we find no significant differences in the values between the two approaches, as shown in Figure 6 where the rates of the R3 reaction are reported. The same comparison of Figure 6 but with reactions R1 and R2 is presented in Supporting Information Section 3, and we find the same degree of numerical agreement between the two approaches, allowing us to conclude that the percentage deviations between the anharmonic coupling values obtained with Gaussian 16 and our implementation are irrelevant for the SCTST reaction rate results.

### 4.1 Reaction Rate Constant Calculations

The reactions of Figure 4 have been previously studied by Greer et al.^68^ In their work, they employed as their top-notch method the small curvature tunneling (SCT) approach.^69^ In their work they also considered the application of monodimensional tunneling corrections, such as Wigner’s and Bell’s corrections, and applied them to the results obtained with the canonical variational theory (CVT) at the B3LYP/6-31G* level of theory. A good agreement between all the methods was found for temperatures above 250 K, and they suggested that there is a significant tunneling contribution for temperatures up to 420 K. They estimated the HAT contribution to be ≥25%. However, a more precise ab initio level of accuracy is necessary before drawing any conclusion about the presence of HAT at room temperatures.

In the present work, we start from the Greer et al.^68^ transition state (TS) geometries, and we optimize them at the level of theory of our anharmonic constants calculation. For each reaction, we double-checked each TS geometry with an intrinsic reaction coordinate (IRC) calculation starting from our TS, obtaining the reactant and product geometries. Geometry details can be found in Supporting Information Section 1.

The TST reaction rate constants are calculated using the following formula
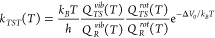
31where Δ*V*_0_ is the difference in energy between the TS and the reactant with the inclusion of the harmonic zero point energy (ZPE) and the rotational *Q*_*R*_^*rot*^(*T*) and *Q*_*TS*_^*rot*^(*T*) partition functions are calculated using eq 9, while the vibrational partition functions are calculated with the harmonic approximation
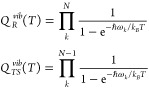
32Since we deal with unimolecular reactions, *N* is the vibrational normal mode number of both the reactant and the TS. For the TS, we consider that the *N*th mode is the imaginary frequency one. In the first formula of eq 32, ω_*k*_ is the vibrational frequency of the *k*th mode of the reactant, while in the second one it is the TS frequency. In eq 31, we do not indicate the translational partition function ratio because the reactions here considered are unimolecular. To estimate the tunneling contribution we define the following general parameter
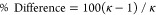
33where the transmission coefficient κ is the ratio between the SCTST rate constant computed using eq 8, which includes tunneling and anharmonic partition function contributions, and the TST one from eq 31, which does not include tunneling contributions and which is strictly harmonic
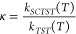
34Before getting into the details of the rate values, we looked for an experimental validation of our computational setup. Specifically, we compare with the experimental results of the reaction barrier estimate for the R1 reaction obtained at different pressures and in a small temperature range.^70^ The comparison is done with the caveat that the experimental values are obtained by enforcing the Arrhenius relation *k*(*T*) ∝ exp (−*E*_*a*_/*k*_*B*_*T*) to the experimental kinetic constants, where *E*_*a*_ is the empirical activation energy barrier. Instead, our barrier estimates Δ*V*_0_ is the energy difference between the TS and the reactants, both corrected for the harmonic ZPE values.

The values of Table 1 show that the computational results at different levels of theory are not within the experimental error bar interval of confidence. Nevertheless, all experimental and computational values are similar and show an overall good agreement, which validates our ab initio setup.

**Table 1:** ZPE Corrected Forward Reaction Barriers for the Cyclobutene Ring Opening Reaction^a^

level of theory/basis set	*E*_*a*_ [kcal/mol]
B3LYP/6-31G*	33.9
MP2/aug-cc-pVDZ	31.2
CCSD(T)/aug-cc-pVDZ	31.7
experiment (8–14 Torr)	32.5 ± 0.5
experiment (100 Torr)	32.5 ± 0.4
experiment (1500 Torr)	32.9 ± 0.7
experiment (5 Torr)	32.7 ± 0.2

a The experimental values^70^ have been calculated at different pressure conditions and in the temperature range 403–448 K.

The values of the forward reaction barrier used to compute the SCTST rate constants include the anharmonic ZPE correction and the  term. This term, although small, can be important in reaction rate calculations, and in our cases it has been determined to be relevant, expecially when evaluating the percentage difference with respect to the TST value. The values of the forward barriers corrected for both anharmonic ZPE and  can be found in Supporting Information Section 4.

Before presenting our rate calculation results, we specify how we deal with hindered rotations (HRs). An accurate HR treatment in the partition functions is of fundamental importance for the calculation of reaction rate constants. We identify the HR degrees of freedom using the Ayala schemes^71^ implemented in the Gaussian 16 software. After the identification and before the VPT2 calculation, the HR modes are projected out from the Hessian matrix, and their one-dimensional partition functions are calculated. For the 1D HR partition functions, we used the Pitzer and Gewin correction to the quantum harmonic oscillator partition function.^72^ With this correction, we can smoothly tune the HR mode treatment from the quantum harmonic oscillator to the free rotor partition function, as the temperature is increased.

**Figure 7:**
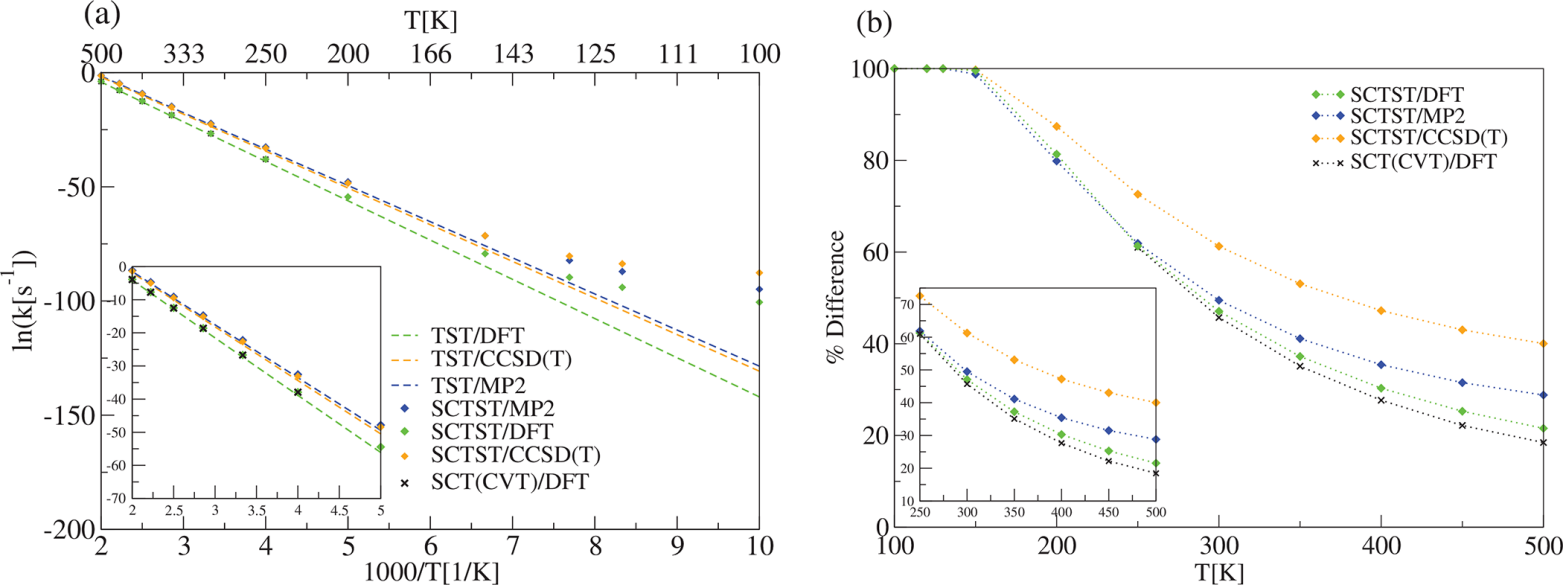
Cyclobutene ring opening reaction (R1). (a) TST, SCTST, and SCT(CVT)^68^ reaction rate constants. The *y* axis reports the natural logarithm of the rate constants and the abscissa the scaled inverse temperature in Kelvin. (b) TST, SCTST, and SCT percentage difference between the semiclassical and the TST kinetic rate constant according to eq 33. For the SCTST rate constant calculations, the anharmonic constant matrix was calculated using our program. DFT calculations have been carried out using B3LYP/6-31G*, while in the MP2 and CCSD(T) calculations we used the aug-cc-pVDZ basis set.

The results for the cyclobutene ring opening reaction R1 of Figure 4 are shown in Figure 7, where panel (a) reports the rate values. An evident bias in the rate estimate is represented by the ab initio theory level, even if post-HF methods give comparable results. These differences should be ascribed mainly to the barrier height, since the exponential function of the rate expression greatly magnifies this dependency. However, when choosing the DFT level of theory, our SCTST results obtained using eq 8 are in close agreement with previous literature results, as highlighted in the inset of panel (a) in Figure 7. In the same panel, a significant deviation from the Arrhenius behavior is observed below *T* = 150 K. We observe also that a significant role in the rate estimate is played by the partition function and by the shape of the barrier. Specifically, in Table 1 the MP2 barrier is lower than the CCSD(T) one, while in Figure 7 the CCSD(T) rate is greater than the MP2 one in the tunneling regime. This is due to a thicker MP2 barrier with respect to the CCSD(T) one (see Supporting Information Section 4). The deviation from Arrhenius linearity is more evident from panel (b) of Figure 7, where the percentage difference with respect to the TST rate is estimated using eq 33. Even at *T* = 500 K an amount of 20% rate enhancement is observed, and we think this is mainly due to the presence of tunneling.

**Figure 8:**
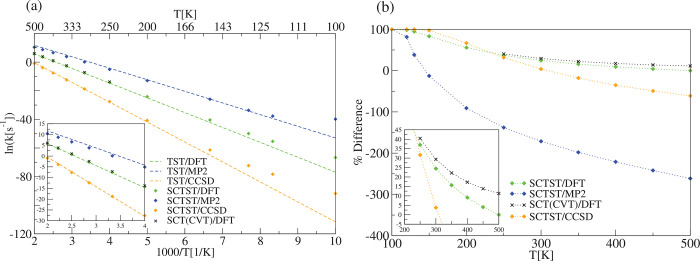
Same as in Figure 7 but for the cis-1,3,5-hexatriene electrocyclic ring closing reaction (R2).

The results for the *cis*-1,3,5-hexatriene electrocyclic ring closing reaction, R2, are shown in Figure 8. In both SCTST and TST calculations, we accounted for two HRs in the reactant molecule. The corresponding frequencies and the Pitzer and Gewin parameters employed can be found in Supporting Information Section 5. Figure 8 shows quite different kinetic rate constants depending on the level of ab intitio theory, which goes from the B3LYP/6-31G* to the post-HF MP2/aug-cc-pVDZ and CCSD/jun-cc-pVDZ level of theory. The values of the energy barriers and the TS frequencies can be found in Supporting Information Section 4. All the rate constant calculations show a certain amount of tunneling and a significant deviation from the Arrhenius linear behavior. In particular, the SCTST rates calculated with the DFT potential once again agree quite well with the SCT(CVT) estimates, as shown in the inset of panel (a) in Figure 8. In panel (b), the percentage difference of each semiclassical rate calculation with respect to the TST one is reported. At low temperatures, we observe for all the semiclassical calculations an almost 100% difference, and we attribute this to tunneling contributions. In the high temperature regime, we think that the anharmonicity of the partition function plays an important role. Specifically, the MP2 barrier is excessively flat and anharmonic if compared to the CCSD and DFT ones, as can be deduced from the higher MP2 rates at any temperature. This flatness generates a significant difference in the TS partition function estimates when using the anharmonic expression of eq 8 versus the harmonic one of eq 32, and it is responsible for the negative percentage differences observed in panel (b) of Figure 8. The higher the temperature, the greater the effect of the potential anharmonicity. A higher level of calculation, such as CCSD, confirms that the large negative percentage difference of the MP2 calculations is due to the limitation of the MP2 level of electronic structure theory.

**Figure 9:**
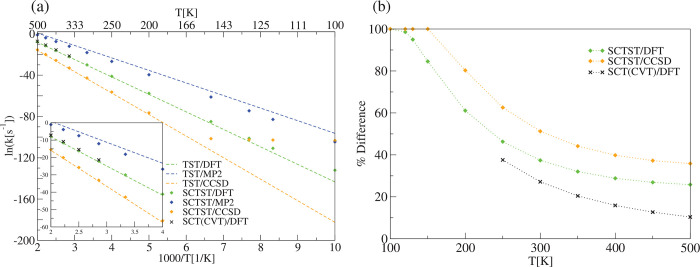
[1,5]-Cope rearrangement reaction of the 1,5-hexadiene molecule (R3). (a) TST, SCTST, and SCT(CVT) reaction rate constants. (b) TST, SCTST, and SCT percentage difference with respect to the TST of the kinetic rate constant. For the SCTST rate constant calculations, the anharmonic constant matrix was calculated using our program. DFT calculations have been carried out using B3LYP/6-31G*, MP2 calculations using the aug-cc-pVDZ basis set, and CCSD calculations using the jun-cc-pVDZ basis set.

The results for the [1,5]-Cope rearrangement reaction of 1,5-hexadiene, the R3 reaction in Figure 4, are shown in Figure 9. Three hindered rotations were considered for the reactant molecule, corresponding to the three normal modes with the lowest frequencies. The results show a strong dependence of the rate constant on the ab initio method used, such as for the R1 and R2 reactions. Specifically, Houk and co-workers^65^ showed that extra care should be taken when dealing with pericyclic reaction ab initio calculations, since correlation contributions to the total energy are large and important. To summarize, they concluded after several benchmark calculations of the forward reaction barriers that the MP2 method probably overestimates the correlation energies and underestimates the barrier height. Indeed, we find in our calculations reported in Figure 9a that the post-HF method CCSD/jun-cc-pVDZ and MP2/aug-cc-pVDZ rates differ by several orders of magnitude. More specifically, the shape of the MP2 potential is so anomalous that the effective imaginary frequecy Ω_*N*_ of eq 7 becomes negative. In this case, the Multiwell program^73^ performs the standard TST calculation; thus, we obtain the Arrhenius plot with blue diamonds reported in Figure 9a. As already observed in the other reactions, the R3 rates shown in the inset of Figure 9a confirm the previous SCT(CVT) results.^68^ Considering the percentage difference between the semiclassical and TST results (see panel (b) of Figure 9), we think that this difference is mainly due to tunneling, especially at low temperatures. In this case, the partition function anharmonicity is not as important as in the case of R2, since negative percentage differences are not observed. At *T* < 150 K, tunneling becomes the only possible way to get to the product side. This is evident also from panel (a) of Figure 9, where the CCSD rate reaches a plateau. In addition, panel (b) shows how CCSD results predict a larger amount of tunneling than the DFT ones, and that in this case there is a slight difference between the amount of tunneling introduced by the SCT(CVT) approach and our SCTST/DFT one.

## 5 Summary and Conclusions

In this paper, we calculate at the CCSD(T) and CCSD level of accuracy tunneling rates involving heavy atoms in organic reactions thanks to our fast and efficient algorithm for the computation of anharmonic constants. Our program can be easily interfaced with any ab initio code available, since only SPE calculations are requested. We use the anharmonic constants as input for the semiclassical transition state theory (SCTST) programs Paradensum and Parsctst^41,42^ from the Multiwell program suite.^38^

First, we tested the performances of our algorithm, showing that it successfully overcomes the limitations of other software. Then, we have tested our program for the SCTST calculation of reaction rate constants on organic reactions with relevant HAT tunneling contribution even at room temperature, as anticipated by Greer et al.^68^ with DFT level of theory estimates. Our code allowed us to extend the HAT phenomena investigation at the post-HF level of theory, up to the CCSD(T) gold standard with consistent basis sets of the type of aug-cc-pVDZ. By varying from the DFT to the MP2 and CCSD(T) or CCSD approaches, we notice conspicuous variations in the reaction rate constants, the tunneling and partition function percentage contributions showing how important the possibility to employ a high level of ab initio theory is. At the B3LYP/6-31G* level, the SCTST approach and the SCT gave similar results for the R1 and R2 reactions, while a slight difference is found for the R3 reaction. We conclude that the use of accurate ab initio post-HF methods is necessary in order to get a reliable PES and anharmonic constants, so that the best possible SCTST reaction rate constant can be calculated.

As far the scaling performances of the code are concerned, we note that the Gaussian 16 software does not actually calculate all the third and fourth order derivatives of the potential but cleverly exploits the symmetry of the molecule to spot zero anharmonicity values and avoid their numerical calculation. We did not implement this symmetry driven strategy in our approach. Nevertheless, the performance of our program overcomes the Gaussian 16 one given the same computational power. A future inclusion of a symmetry tool in our algorithm may be useful to further reduce the computational time, especially for calculations on organic molecules of biological interest.

Gaussian 16^52^ employs the Thiel et al.^74^ scheme where the gradients are calculated analytically and then the Hessians are differentiated numerically to obtain the needed third and fourth order potential derivatives. This guarantees a higher precision with respect to our approach where the finite differences of the energy are employed. However, as shown above, the scaling and the parallelization efficiency of Gaussian 16 is worse than in our implementation, and the loss of accuracy in estimating the anharmonic couplings with our procedure leads to negligible differences in the rate constant values.

We recall here that couple cluster parallel calculations of anharmonic constants can also be performed with CFOUR ab initio software^75^ by setting up an appropriate procedure as explained in the CFOUR user manual.^76^ The algorithm presented in our work is a possible alternative to that implementation. Depending on the computational efficiency in parallel architectures of these calculations, one should choose which approach to adopt by estimating the trade-off between computational time and accuracy.

We think the strengths of our code are the high level of automation and that it can be interfaced with any quantum chemistry packages, allowing the user to choose among diverse ab initio methods. In any case, our code is readily modifiable. For instance, it is possible to change the schemes adopted to calculate the anharmonic constants. In the present paper we used a 3MR-QFF for the calculation of the anharmonic constants, but it is possible to further reduce the number of coupled modes in the QFF representation of the potential and find new formulas for the anharmonic constants. For example, the 2MR representation opens the possibility to an alternative approximation of the SCTST rate constants, different from the reduced dimensionality approaches present in the literature.^77^ We will further study the implications of the 2MR approximation in the SCTST treatment.

Our algorithm and the related scripts will be made available as part of the Multiwell suite of codes.^38^

## Appendix A

To rewrite the potential expansion, we employ the nMR as suggested by Yagi et al.^59^ Mathematically this corresponds to a rearrangement of the Taylor expansion of the potential by writing the *k*-order differential form in a more concise form using a multi-index formulation. In the case of a generic differentiable function *f*, we define α = (α_1_, ..., α_*n*_), |α| = α_1_ + ... + α_*n*_, α! = α_1_!...α_*n*_!, and, given a vector **x**, **x**^α^ = *x*_1_^α_1_^...*x*_*n*_^α_*n*_^. In this formulation |α| is called “height of the multi-index α”, and the |α| order derivative of the *f* function is given by

35

Using this notation we can write

36

In this way, the generic *f* Taylor expansion can be written as

37

## Appendix B

The diagonal elements of the tensor are calculated according to the following formula

38

and the off-diagonal elements are

39

## Appendix C

For the 1MR-QFF derivatives we used the central approximation at a grid point (+3, + 2, ..., −3) with four orders of accuracy

40

For the 2MR-QFF third derivatives *f*_*iij*_, we combined the central second order derivative scheme at grid points (+1, 0, −1) along the mode *i* with the central first derivative scheme of order 2 in the same points (+1, 0, −1), and we obtained the following expression

41

For the fourth order derivative *f*_*iiij*_ we combined the central first derivative scheme of order 2 at the grid points (+1, 0, −1) along *j* and the centered approximation at the halfway points (−3/2, −1/2, +1/2, +3/2) for the third order derivative along *i*:

42

For the 3MR-QFF third order derivative *f*_*ijk*_, we used the central first derivative scheme of order 2 at the grid points (+1, 0, −1) for each normal mode, and we obtained:

43

For the fourth order derivative *f*_*iijk*_, we used the central first derivative scheme of order 2 at the grid points (+1, 0, −1) along the modes *j* and *k* and the central second order derivative along the mode *i* of order 2 of approximation at the grid points (+1, 0, −1):

44

## Supporting Information Available

Electronic structure calculations and all the reactant and transition state optimized geometries; additional scalability and accuracy tests, detailed discussion of the energetics of the studied reactions, hindered rotational mode (HRM) treatment involved in the low frequency modes of the R2 and R3 reactants, and finally all the caluculated kinetic constants in table form (PDF)

## References

[ref1] Miller W. H. *Direct* and *correct* calculation of canonical and microcanonical rate constants for chemical reactions. J. Phys. Chem. A 1998, 102, 793–806. 10.1021/jp973208o.

[ref2] Berne B. J.; Ciccotti G.; Coker D. F. Classical and Quantum Dynamics in Condensed Phase Simulations; World Scientific: 1998.

[ref3] Chandler D. Roles of classical dynamics and quantum dynamics on activated processes occurring in liquids. J. Stat. Phys. 1986, 42, 49–67. 10.1007/BF01010840.

[ref4] Miller W. H.; Schwartz S. D.; Tromp J. W. Quantum mechanical rate constants for bimolecular reactions. J. Chem. Phys. 1983, 79, 4889–4898. 10.1063/1.445581.

[ref5] Park T. J.; Light J. Quantum flux operators and thermal rate constant: Collinear *H* + *H*_2_. J. Chem. Phys. 1988, 88, 4897–4912. 10.1063/1.454702.

[ref6] Day P. N.; Truhlar D. G. Calculation of thermal rate coefficients from the quantum flux autocorrelation function: Converged results and variational quantum transition state theory for *O* + *HD* ↔ *OD* + *H* and *O* + *HD* ↔ *OH* + *D*. J. Chem. Phys. 1991, 95, 5097–5112. 10.1063/1.461677.

[ref7] Wang H.; Thompson W. H.; Miller W. H. Thermal rate constant calculation using flux-flux autocorrelation functions: Application to *Cl* + *H*_2_ → *HCl* + *H* reaction. J. Chem. Phys. 1997, 107, 7194–7201. 10.1063/1.474959.

[ref8] Tromp J. W.; Miller W. H. The reactive flux correlation function for collinear reactions *H* + *H*_2_*Cl* + *HCl* and *F* + *H*_2_. Faraday Discuss. Chem. Soc. 1987, 84, 441–453. 10.1039/dc9878400441.

[ref9] Thompson W. H.; Miller W. H. On the direct calculation of thermal rate constants. II. The flux-flux autocorrelation function with absorbing potentials, with application to the *O* + *HCl* → *OH* + *Cl* reaction. J. Chem. Phys. 1997, 106, 142–150. 10.1063/1.474109.

[ref10] Manthe U.; Matzkies F. Quantum calculations of thermal rate constants and reaction probabilities: *H*_2_ + *CN* → *H* + *HCN*. Chem. Phys. Lett. 1998, 282, 442–449. 10.1016/S0009-2614(97)01236-0.

[ref11] Matzkies F.; Manthe U. Accurate quantum calculations of thermal rate constants employing MCTDH: *H*_2_ + *OH* → *H* + *H*_2_*O* and *D*_2_ + *OH* → *D* + *DOH*. J. Chem. Phys. 1998, 108, 4828–4836. 10.1063/1.475892.

[ref12] Mandrà S.; Valleau S.; Ceotto M. Deep nuclear resonant tunneling thermal rate constant calculations. Int. J. Quantum Chem. 2013, 113, 1722–1734. 10.1002/qua.24395.

[ref13] Eyring H. The activated complex in chemical reactions. J. Chem. Phys. 1935, 3, 107–115. 10.1063/1.1749604.

[ref14] Wigner E. Calculation of the rate of elementary association reactions. J. Chem. Phys. 1937, 5, 720–725. 10.1063/1.1750107.

[ref15] Wigner E. Über das Überschreiten von Potentialschwellen bei chemischen Reaktionen. Z. Phys. Chem. 1932, 19B, 203–216. 10.1515/zpch-1932-1920.

[ref16] Eckart C. The penetration of a potential barrier by electrons. Phys. Rev. 1930, 35, 1303–1309. 10.1103/PhysRev.35.1303.

[ref17] Zaverkin V.; Kästner J. Instanton Theory to Calculate Tunnelling Rates and Tunnelling Splittings. In Tunnelling in Molecules; Royal Society of Chemistry: 2020; pp 245–260.

[ref18] Bao J. L.; Truhlar D. G. Variational transition state theory: theoretical framework and recent developments. Chem. Soc. Rev. 2017, 46, 7548–7596. 10.1039/C7CS00602K. 29165460

[ref19] Craig I. R.; Manolopoulos D. E. Chemical reaction rates from ring polymer molecular dynamics. J. Chem. Phys. 2005, 122, 084106 10.1063/1.1850093. 15836019

[ref20] Richardson J. O. Ring-polymer instanton theory. Int. Rev. Phys. Chem. 2018, 37, 171–216. 10.1080/0144235X.2018.1472353. 29865828

[ref21] Aieta C.; Ceotto M. A quantum method for thermal rate constant calculations from stationary phase approximation of the thermal flux-flux correlation function integral. J. Chem. Phys. 2017, 146, 214115 10.1063/1.4984099. 28595397

[ref22] Karandashev K.; Vaníček J. Accelerating quantum instanton calculations of the kinetic isotope effects. J. Chem. Phys. 2015, 143, 194104 10.1063/1.4935701. 26590524

[ref23] Mandrà S.; Schrier J.; Ceotto M. Helium isotope enrichment by resonant tunneling through nanoporous graphene bilayers. J. Phys. Chem. A 2014, 118, 6457–6465. 10.1021/jp502548r. 24854987

[ref24] Ceotto M. Vibration-assisted tunneling: a semiclassical instanton approach. Mol. Phys. 2012, 110, 547–559. 10.1080/00268976.2012.663943.

[ref25] Miller W. H.; Zhao Y.; Ceotto M.; Yang S. Quantum instanton approximation for thermal rate constants of chemical reactions. J. Chem. Phys. 2003, 119, 1329–1342. 10.1063/1.1580110.

[ref26] Ceotto M.; Miller W. H. Test of the quantum instanton approximation for thermal rate constants for some collinear reactions. J. Chem. Phys. 2004, 120, 6356–6362. 10.1063/1.1666064. 15267524

[ref27] Ceotto M.; Yang S.; Miller W. H. Quantum reaction rate from higher derivatives of the thermal flux-flux autocorrelation function at time zero. J. Chem. Phys. 2005, 122, 044109 10.1063/1.1839177. 15740237

[ref28] Larrégaray P.; Bonnet L. Including tunneling into the classical cross sections and rate constants for the *N* (^2^*D*) + *H*_2_ (*v* = 0, *j* = 0) reaction. Theor. Chem. Acc. 2021, 140, 61 10.1007/s00214-021-02749-6.

[ref29] Miller W. H. Semiclassical limit of quantum mechanical transition state theory for nonseparable systems. J. Chem. Phys. 1975, 62, 1899–1906. 10.1063/1.430676.

[ref30] Miller W. H. Semi-classical theory for non-separable systems:. Construction of good action-angle variables for reaction rate constants. Faraday Discuss. Chem. Soc. 1977, 62, 40–46. 10.1039/DC9776200040.

[ref31] Miller W. H.; Hernandez R.; Handy N. C.; Jayatilaka D.; Willetts A. Ab initio calculation of anharmonic constants for a transition state, with application to semiclassical transition state tunneling probabilities. Chem. Phys. Lett. 1990, 172, 62–68. 10.1016/0009-2614(90)87217-F.

[ref32] Hernandez R.; Miller W. H. Semiclassical transition state theory. A new perspective. Chem. Phys. Lett. 1993, 214, 129–136. 10.1016/0009-2614(93)90071-8.

[ref33] Nguyen T. L.; Stanton J. F.; Barker J. R. A practical implementation of semi-classical transition state theory for polyatomics. Chem. Phys. Lett. 2010, 499, 9–15. 10.1016/j.cplett.2010.09.015.

[ref34] Nguyen T. L.; Barker J. R.; Stanton J. F. Atmospheric Reaction Rate Constants and Kinetic Isotope Effects Computed Using the HEAT Protocol and Semi-Classical Transition State Theory. In Advances in Atmospheric Chemistry; World Scientific: 2016; pp 403–492.

[ref35] Nguyen T. L.; Stanton J. F.; Barker J. R. Ab initio reaction rate constants computed using semiclassical transition-state theory: *HO* + *H*_2_ → *H*_2_*O* + *H* and isotopologues. J. Phys. Chem. A 2011, 115, 5118–5126. 10.1021/jp2022743. 21539339

[ref36] Wagner A. F. Improved multidimensional semiclassical tunneling theory. J. Phys. Chem. A 2013, 117, 13089–13100. 10.1021/jp409720s. 24224758

[ref37] Stanton J. F. Semiclassical transition-state theory based on fourth-order vibrational perturbation theory: The symmetrical eckart barrier. J. Phys. Chem. Lett. 2016, 7, 2708–2713. 10.1021/acs.jpclett.6b01239. 27358083

[ref38] Barker J. R.; Nguyen T. L.; Stanton J. F.; Aieta C.; Ceotto M.; Gabas F.; Kumar T. J. D.; Li C.; Lohr L. L.; Maranzana A.; Ortiz N. F.; Preses J. M.; Simmie J. M.; Sonk J. A.; Stimac P. J. MultiWell-2020 Software Suite; University of Michigan: Ann Arbor, Michigan, U.S.A., 2020. http://clasp-research.engin.umich.edu/multiwell/.

[ref39] Barker J. R. Multiple-Well, multiple-path unimolecular reaction systems. I. MultiWell computer program suite. Int. J. Chem. Kinet. 2001, 33, 232–245. 10.1002/kin.1017.

[ref40] Barker J. R. Energy transfer in master equation simulations: A new approach. Int. J. Chem. Kinet. 2009, 41, 748–763. 10.1002/kin.20447.

[ref41] Aieta C.; Gabas F.; Ceotto M. An efficient computational approach for the calculation of the vibrational density of states. J. Phys. Chem. A 2016, 120, 4853–4862. 10.1021/acs.jpca.5b12364. 26840098

[ref42] Aieta C.; Gabas F.; Ceotto M. Parallel Implementation of Semiclassical Transition State Theory. J. Chem. Theory Comput. 2019, 15, 2142–2153. 10.1021/acs.jctc.8b01286. 30822385

[ref43] Greene S. M.; Shan X.; Clary D. C. Rate constants of chemical reactions from semiclassical transition state theory in full and one dimension. J. Chem. Phys. 2016, 144, 244116 10.1063/1.4954840. 27369506

[ref44] Greene S. M.; Shan X.; Clary D. C. Quantum Scattering and Semiclassical Transition State Theory Calculations on Chemical Reactions of Polyatomic Molecules in Reduced Dimensions. Adv. Chem. Phys. 2018, 163, 117–149. 10.1002/9781119374978.ch4.

[ref45] Shan X.; Burd T. A.; Clary D. C. New developments in semiclassical transition-state theory. J. Phys. Chem. A 2019, 123, 4639–4657. 10.1021/acs.jpca.9b01987. 30969125

[ref46] Barnes L.; Schindler B.; Compagnon I.; Allouche A.-R. iGVPT2: an interface to computational chemistry packages for anharmonic corrections to vibrational frequencies. arXiv, 2017; arXiv:physics.chem-ph/1704.02144 (accessed Oct. 1, 2019).

[ref47] Miller W. H. Semiclassical limit of quantum mechanical transition state theory for nonseparable systems. J. Chem. Phys. 1975, 62, 1899–1906. 10.1063/1.430676.

[ref48] Miller W. H. Quantum mechanical transition state theory and a new semiclassical model for reaction rate constants. J. Chem. Phys. 1974, 61, 1823–1834. 10.1063/1.1682181.

[ref49] Gutzwiller M. C. Periodic orbits and classical quantization conditions. J. Math. Phys. 1971, 12, 343–358. 10.1063/1.1665596.

[ref50] Fröman N.; Fröman P. O. JWKB approximation: contributions to the theory; North-Holland: 1965.

[ref51] Mills I. M. In Molecular spectroscopy: modern research; Rao K. N., Mathews C. W., Eds.; Academic Press: New York, 1972; pp 115–140.

[ref52] Barone V. Anharmonic vibrational properties by a fully automated second-order perturbative approach. J. Chem. Phys. 2005, 122, 014108 10.1063/1.1824881. 15638643

[ref53] Rosnik A. M.; Polik W. F. VPT2+ K spectroscopic constants and matrix elements of the transformed vibrational Hamiltonian of a polyatomic molecule with resonances using Van Vleck perturbation theory. Mol. Phys. 2014, 112, 261–300. 10.1080/00268976.2013.808386.

[ref54] Dunham J. The energy levels of a rotating vibrator. Phys. Rev. 1932, 41, 721 10.1103/PhysRev.41.721.

[ref55] Watson J. K. Simplification of the molecular vibration-rotation Hamiltonian. Mol. Phys. 1968, 15, 479–490. 10.1080/00268976800101381.

[ref56] Eckart C. Some Studies Concerning Rotating Axes and Polyatomic Molecules. Phys. Rev. 1935, 47, 552–558. 10.1103/PhysRev.47.552.

[ref57] Wilson E. B. Jr; Howard J. The Vibration-Rotation Energy Levels of Polyatomic Molecules I. Mathematical Theory of Semirigid Asymmetrical Top Molecules. J. Chem. Phys. 1936, 4, 260–268. 10.1063/1.1749833.

[ref58] Meal J. H.; Polo S. Vibration-Rotation Interaction in Polyatomic Molecules. I. The Zeta Matrices. J. Chem. Phys. 1956, 24, 1119–1125. 10.1063/1.1742728.

[ref59] Yagi K.; Hirao K.; Taketsugu T.; Schmidt M. W.; Gordon M. S. Ab initio vibrational state calculations with a quartic force field: Applications to *H*_2_*CO*, *C*_2_*H*_4_, *CH*_3_*OH*, *CH*_3_*CCH*, and *C*_6_*H*_6_. J. Chem. Phys. 2004, 121, 1383–1389. 10.1063/1.1764501. 15260682

[ref60] Fornberg B. Generation of finite difference formulas on arbitrarily spaced grids. Math. Comp. 1988, 51, 699–706. 10.1090/S0025-5718-1988-0935077-0.

[ref61] Martin J. M.; Lee T. J.; Taylor P. R.; François J.-P. The anharmonic force field of ethylene, *C*_2_*H*_4_, by means of accurate ab initio calculations. J. Chem. Phys. 1995, 103, 2589–2602. 10.1063/1.469681.

[ref62] Frisch M. J. Gaussian 03; Gaussian, Inc.: Wallingford, CT, 2004.

[ref63] GALILEO IBM NeXtScale cluster. https://www.hpc.cineca.it/hardware/galileo (accessed January 27, 2021).

[ref64] Castro C.; Karney W. L. Heavy-Atom Tunneling in Organic Reactions. Angew. Chem., Int. Ed. 2020, 59, 8355–8366. 10.1002/anie.201914943. 31944500

[ref65] Guner V.; Khuong K. S.; Leach A. G.; Lee P. S.; Bartberger M. D.; Houk K. A standard set of pericyclic reactions of hydrocarbons for the benchmarking of computational methods: the performance of ab initio, density functional, CASSCF, CASPT2, and CBS-QB3 methods for the prediction of activation barriers, reaction energetics, and transition state geometries. J. Phys. Chem. A 2003, 107, 11445–11459. 10.1021/jp035501w.

[ref66] Sarkar S. K.; Solel E.; Kozuch S.; Abe M. Heavy-Atom Tunneling Processes during Denitrogenation of 2,3-Diazabicyclo[2.2.1]hept-2-ene and Ring Closure of Cyclopentane-1,3-diyl Diradical. Stereoselectivity in Tunneling and Matrix Effect. J. Org. Chem. 2020, 85, 8881–8892. 10.1021/acs.joc.0c00763. 32527076

[ref67] Carpenter B. K. Heavy-atom tunneling as the dominant pathway in a solution-phase reaction? Bond shift in antiaromatic annulenes. J. Am. Chem. Soc. 1983, 105, 1700–1701. 10.1021/ja00344a073.

[ref68] Doubleday C.; Armas R.; Walker D.; Cosgriff C. V.; Greer E. M. Heavy-Atom Tunneling Calculations in Thirteen Organic Reactions: Tunneling Contributions are Substantial, and Bell’s Formula Closely Approximates Multidimensional Tunneling at *T* > 250 K. Angew. Chem., Int. Ed. 2017, 129, 13279–13282. 10.1002/ange.201708489. 28881399

[ref69] Liu Y. P.; Lynch G. C.; Truong T. N.; Lu D. H.; Truhlar D. G.; Garrett B. C. Molecular modeling of the kinetic isotope effect for the [1, 5]-sigmatropic rearrangement of cis-1, 3-pentadiene. J. Am. Chem. Soc. 1993, 115, 2408–2415. 10.1021/ja00059a041.

[ref70] Lewis K.; Steiner H. 588. The kinetics and mechanism of the thermal cyclisation of hexa-1, cis-3,5-triene to cyclohexa-1,3-diene. J. Chem. Soc. 1964, 3080–3092. 10.1039/jr9640003080.

[ref71] Ayala P. Y.; Schlegel H. B. Identification and treatment of internal rotation in normal mode vibrational analysis. J. Chem. Phys. 1998, 108, 2314–2325. 10.1063/1.475616.

[ref72] Pitzer K. S.; Gwinn W. D. Energy levels and thermodynamic functions for molecules with internal rotation I. Rigid frame with attached tops. J. Chem. Phys. 1942, 10, 428–440. 10.1063/1.1723744.

[ref73] Nguyen T. L.; Barker J. R.; Stanton J. F. Advances in Atmospheric Chemistry; World Scientific, 2017; pp 403–492.

[ref74] Schneider W.; Thiel W. Anharmonic force fields from analytic second derivatives: Method and application to methyl bromide. Chem. Phys. Lett. 1989, 157, 367–373. 10.1016/0009-2614(89)87263-X.

[ref75] Matthews D. A.; Cheng L.; Harding M. E.; Lipparini F.; Stopkowicz S.; Jagau T.-C.; Szalay P. G.; Gauss J.; Stanton J. F. Coupled-cluster techniques for computational chemistry: The CFOUR program package. J. Chem. Phys. 2020, 152, 214108 10.1063/5.0004837. 32505146

[ref76] CFOUR user manual. http://slater.chemie.uni-mainz.de/cfour/index.php?n=Main.CalculatingHarmonicFrequenciesByFiniteDifferencesInParallel (accessed November 2, 2021).

[ref77] Greene S. M.; Shan X.; Clary D. C. An investigation of one- versus two-dimensional semiclassical transition state theory for H atom abstraction and exchange reactions. J. Chem. Phys. 2016, 144, 084113 10.1063/1.4942161. 26931687

