# Supplementary material for: Heavy Atom Tunneling in Organic Reactions at Coupled Cluster Potential Accuracy with a Parallel Implementation of Anharmonic Constant Calculations and Semiclassical Transition State Theory
Source: J Chem Theory Comput. 2022 Jan 7;18(2):623–37. doi: 10.1021/acs.jctc.1c01143 (PMC8830048; doi:10.1021/acs.jctc.1c01143)
Supplement: Supplementary file 1 — ct1c01143_si_001.pdf [file ct1c01143_si_001.pdf]

Supporting Information: Heavy Atom Tunneling in Organic Reactions at Coupled Cluster Potential Accuracy with a Parallel Implementation of Anharmonic Constant Calculations and Semiclassical Transition State Theory

Giacomo Mandelli,[†,‡] Chiara Aieta,[†,‡] and Michele Ceotto[*,†]

†*Dipartimento di Chimica, Università degli Studi di Milano, via C. Golgi 19, 20133 Milano, Italy*

‡*These authors contributed equally*

E-mail: michele.ceotto@unimi.it

Contents

1 Electronic Structure Calculations and Optimized Geometries 2

2 Scalability Tests 8

3 Accuracy Tests 9

4 Energetic of the Reactions 19

5 Hindered Rotations Treatment 20

6 Kinetic Rate Constants 23

References 26

1 Electronic Structure Calculations and Optimized Geometries

In our paper, we performed Single Point electronic structure Energy (SPE) calculations and geometry optimizations using the Gaussian16 software.[1]

The geometry optimizations are carried out requiring the lowest possible residual gradient in the optimization process. The following input keywords are set as default in our program for the geometry optimization step:

- SCF=(XQC,Tight) and Int=UltraFine (for DFT calculations)
- Opt=(VeryTight,CalcAll,MaxCycles=200) (add TS option for the transition state opt)

For the CCSD(T) optimization we started from the CCSD optimized geometry, for which we have analytic gradients available. Then we switched to numerical gradients for CCSD(T) calculations with the eigenvalue-following algorithm for the optimization process. A first

calculation is done using the standard setup, with a maximum step size of 0.1 Bohr. Once the Tight convergence is reached, new optimization parameters have been used for the VeryTight convergence criteria. The following overlay 1 options have been used for the R1 reactant molecule:

- IOp(1/8=4), maximum step size of 0.04 Bohr for the optimization
- IOp(1/19=8) Newton-Raphson and linear search
- IOp(1/13=9) BFGS Hessian update

and the same options with a 0.03 Bohr step size for the R1 TS molecule.

The optimized geometries are listed below in Cartesian coordinates for the MP2, DFT and CCSD calculations as well as the Z-matrix for the CCSD(T). All the coordinates are given in Ångström. The R1, R2, R3 notation refers to the one defined in the article in figure 4.

Table 1: R1 optimized geometries at B3LYP/6-31G* and MP2/aug-cc-pVDZ level of theory

(a) R, MP2

H	0.000000	-1.431471	1.613362
C	-0.000000	0.681017	0.816768
C	0.000000	-0.681017	0.816768
C	-0.000000	0.788756	-0.705211
C	-0.000000	-0.788756	-0.705211
H	0.898056	-1.246261	-1.150263
H	-0.898056	-1.246261	-1.150263
H	0.000000	1.431471	1.613362
H	-0.898056	1.246261	-1.150263
H	0.898056	1.246261	-1.150263

(b) TS, MP2

H	1.877113	-1.148110	-0.438674
C	1.063482	-0.654695	0.116257
C	-1.064062	-0.653755	-0.116245
C	0.690129	0.719207	-0.095810
C	-0.689492	0.719821	0.095796
H	-1.878131	-1.146437	0.438696
H	-1.350943	1.527640	0.426355
H	0.834037	-1.101199	1.085773
H	-0.835014	-1.100480	-1.085752
H	1.352296	1.526434	-0.426384

(c) R, DFT/B3LYP

H	0.000000	-1.420403	1.601596
C	-0.000000	0.670184	0.814741
C	0.000000	-0.670184	0.814741
C	-0.000000	0.786267	-0.699538
C	-0.000000	-0.786267	-0.699538
H	0.889920	-1.246838	-1.146204
H	-0.889920	-1.246838	-1.146204
H	0.000000	1.420403	1.601596
H	-0.889920	1.246838	-1.146204
H	0.889920	1.246838	-1.146204

(d) TS, DFT/B3LYP

H	1.882083	-1.124577	-0.435337
C	1.064867	-0.649843	0.119100
C	-1.065442	-0.648900	-0.119085
C	0.683672	0.709195	-0.083820
C	-0.683042	0.709803	0.083807
H	-1.883080	-1.122898	0.435361
H	-1.338893	1.518545	0.403252
H	0.871055	-1.110497	1.080531
H	-0.872040	-1.109746	-1.080507
H	1.340240	1.517349	-0.403282

(e) P, DFT/B3LYP

C	-0.109096	0.000103	1.848685
C	0.400558	0.000068	0.608789
C	-0.400558	0.000037	-0.608788
H	1.482676	0.000061	0.474061
H	-1.482676	0.000044	-0.474061
C	0.109096	0.000002	-1.848685
H	1.183017	-0.000006	-2.022650
H	0.526412	0.000125	2.729077
H	-1.183018	0.000111	2.022650
H	-0.526413	-0.000020	-2.729076

(f) P, MP2

C	-0.110413	0.000103	1.860162
C	0.409433	0.000068	0.607116
C	-0.409433	0.000038	-0.607116
H	1.498651	0.000061	0.474195
H	-1.498651	0.000044	-0.474195
C	0.110412	0.000002	-1.860162
H	1.192995	-0.000006	-2.020119
H	0.533079	0.000125	2.742911
H	-1.192995	0.000111	2.020119
H	-0.533079	-0.000020	-2.742911

Table 2: R1 optimized geometries at CCSD(T)/aug-cc-pVDZ level of theory

(a) R, CCSD(T)

H							
C	1	B1					
C	1	B2	2	A2			
C	2	B3	1	A3	3	D3	0
C	3	B4	1	A4	2	D4	0
H	5	B5	3	A5	1	D5	0
H	5	B6	3	A6	1	D6	0
H	2	B7	1	A7	3	D7	0
H	4	B8	2	A8	1	D8	0
H	4	B9	2	A9	1	D9	0

Variables:	
B1	2.2636
B2	1.097
A2	25.9704
B3	1.5359
A3	114.7223
D3	-0.0002
B4	1.5359
A4	132.4663
D4	180.0001
B5	1.1053
A5	115.5891
D5	295.3521
B6	1.1053
A6	115.589
D6	64.6487
B7	1.097
A7	112.8104
D7	180.0004
B8	1.1053
A8	115.5891
D8	115.3521
B9	1.1053
A9	115.589
D9	244.6487

(b) TS, CCSD(T)

H							
C	1	B1					
C	2	B2	1	A2			
C	2	B3	1	A3	3	D3	0
C	4	B4	2	A4	1	D4	0
H	3	B5	2	A5	1	D5	0
H	5	B6	4	A6	2	D6	0
H	2	B7	1	A7	3	D7	0
H	3	B8	2	A8	1	D8	0
H	4	B9	2	A9	1	D9	0

Variables:	
B1	1.104
B2	2.163
A2	132.4179
B3	1.4501
A3	122.7685
D3	-97.9636
B4	1.3928
A4	104.1571
D4	144.3749
B5	1.104
A5	132.4154
D5	106.7401
B6	1.098
A6	129.5336
D6	154.4381
B7	1.0944
A7	114.6331
D7	106.9788
B8	1.0944
A8	85.1134
D8	-12.5028
B9	1.098
A9	126.1322
D9	-31.1543

(c) P, CCSD(T)

C							
C	1	B1					
C	2	B2	1	A2			
H	2	B3	1	A3	3	D3	0
H	3	B4	2	A4	1	D4	0
C	3	B5	2	A5	1	D5	0
H	6	B6	3	A6	2	D6	0
H	1	B7	2	A7	3	D7	0
H	1	B8	2	A8	3	D8	0
H	6	B9	3	A9	2	D9	0

Variables:	
B1	1.3609
B2	1.4734
A2	123.5227
B3	1.1001
A3	119.6435
D3	180.0007
B4	1.1001
A4	116.8342
D4	0.0002
B5	1.3609
A5	123.5222
D5	180.0003
B6	1.0977
A6	121.0953
D6	-0.0003
B7	1.0957
A7	121.4771
D7	179.9995
B8	1.0977
A8	121.0954
D8	359.9993
B9	1.0957
A9	121.4771
D9	179.9994

Table 3: (Part 1) R2 optimized geometries at B3LYP/6-31G*, MP2/aug-cc-pVDZ and CCSD/jun-cc-pVDZ level of theory

(a) R, MP2

C	-1.144539	1.580279	0.599986
C	-0.014074	1.580910	-0.145523
C	1.132553	0.669393	0.059052
C	1.105216	-0.678990	0.256434
C	-0.065110	-1.581610	0.200895
C	-1.002092	-1.573039	-0.776717
H	0.104563	2.347844	-0.921662
H	2.121210	1.146333	0.062437
H	2.066366	-1.163418	0.471945
H	-0.127343	-2.348760	0.983367
H	-1.825039	-2.291984	-0.772433
H	-0.958198	-0.846767	-1.592578
H	-1.940573	2.305441	0.413946
H	-1.288151	0.854389	1.404652

(b) R, DFT/B3LYP

C	1.65971	-1.09333	-0.41294
C	1.62945	0.05969	0.2674
C	0.66188	1.15916	0.13335
C	-0.66188	1.15916	-0.13335
C	-1.62945	0.05969	-0.2674
C	-1.65971	-1.09333	0.41294
H	2.45336	0.27023	0.95152
H	1.11156	2.14467	0.26383
H	-1.11156	2.14467	-0.26383
H	-2.45336	0.27022	-0.95153
H	-2.46114	-1.81113	0.26166
H	-0.8939	-1.3569	1.13593
H	2.46114	-1.81113	-0.26166
H	0.8939	-1.35691	-1.13593

(c) R, CCSD/jun-cc-pVDZ

C	-1.138302	1.627065	0.628833
C	-0.023081	1.592411	-0.132320
C	1.126319	0.668228	0.071648
C	1.101938	-0.677789	0.242771
C	-0.071052	-1.593053	0.186014
C	-0.989969	-1.619896	-0.803503
H	0.095883	2.336243	-0.934377
H	2.114224	1.152275	0.085986
H	2.064731	-1.169327	0.447428
H	-0.138537	-2.337083	0.993850
H	-1.811988	-2.345205	-0.790324
H	-0.946283	-0.918581	-1.645326
H	-1.931411	2.358577	0.434331
H	-1.287684	0.926156	1.458790

(d) TS, MP2

C	-1.212831	1.136147	-0.110743
C	0.116110	1.481498	0.203290
C	1.251613	0.708474	-0.125272
C	1.251613	-0.708474	-0.125272
C	0.116110	-1.481498	0.203290
C	-1.212831	-1.136147	-0.110743
H	0.287860	2.316791	0.896076
H	2.235936	1.184919	-0.044816
H	2.235936	-1.184919	-0.044816
H	0.287860	-2.316791	0.896076
H	-2.020621	-1.628122	0.446537
H	-1.450297	-0.920735	-1.147908
H	-2.020621	1.628122	0.446537
H	-1.450297	0.920735	-1.147908

(e) TS, DFT/B3LYP

C	-1.203685	1.140779	-0.106145
C	0.119802	1.482612	0.188744
C	1.242310	0.702978	-0.106182
C	1.242310	-0.702978	-0.106182
C	0.119802	-1.482612	0.188744
C	-1.203685	-1.140779	-0.106145
H	0.296293	2.340220	0.841809
H	2.220032	1.175092	-0.016766
H	2.220032	-1.175092	-0.016766
H	0.296293	-2.340220	0.841809
H	-1.999232	-1.641166	0.447018
H	-1.467751	-0.936630	-1.131315
H	-1.999232	1.641166	0.447018
H	-1.467751	0.936630	-1.131315

(f) TS, CCSD/jun-cc-pVDZ

C	-1.211632	1.124331	-0.107417
C	0.118882	1.484638	0.197519
C	1.252491	0.708684	-0.116752
C	1.252491	-0.708684	-0.116752
C	0.118882	-1.484638	0.197519
C	-1.211632	-1.124331	-0.107417
H	0.291166	2.342573	0.865193
H	2.237920	1.185894	-0.028450
H	2.237920	-1.185894	-0.028450
H	0.291166	-2.342573	0.865193
H	-2.019247	-1.618255	0.454315
H	-1.461810	-0.925646	-1.147245
H	-2.019247	1.618255	0.454315
H	-1.461810	0.925646	-1.147245

Table 4: (Part2) R2 optimized geometries at B3LYP/6-31G*, MP2/aug-cc-pVDZ and CCSD/jun-cc-pVDZ level of theory

(a) P, MP2

C	1.426702	0.088132	-0.093129
H	2.510264	0.246382	-0.093261
C	0.722838	0.134715	-1.258504
H	1.228474	0.351742	-2.204801
C	-0.722831	-0.134716	-1.258508
H	-1.228461	-0.351744	-2.204808
C	-1.426702	-0.088133	-0.093137
H	-2.510263	-0.246383	-0.093275
C	-0.716645	0.275583	1.193979
H	-0.685023	1.381124	1.281898
H	-1.271664	-0.103120	2.066951
C	0.716637	-0.275583	1.193983
H	0.685015	-1.381124	1.281902
H	1.271651	0.103121	2.066959

(b) P, DFT/B3LYP

C	1.425046	0.064445	-0.113679
H	2.506220	0.185099	-0.115735
C	0.726173	0.104203	-1.260201
H	1.227139	0.272675	-2.210923
C	-0.726166	-0.104204	-1.260205
H	-1.227126	-0.272676	-2.210931
C	-1.425046	-0.064446	-0.113687
H	-2.506220	-0.185100	-0.115750
C	-0.731549	0.239765	1.195321
H	-0.762431	1.330411	1.361316
H	-1.272151	-0.208877	2.037036
C	0.731541	-0.239765	1.195325
H	0.762423	-1.330411	1.361321
H	1.272139	0.208877	2.037043

(c) P, CCSD/jun-cc-pVDZ

C	1.430623	0.078158	-0.103671
H	2.518668	0.220737	-0.103296
C	0.731163	0.119921	-1.264847
H	1.236753	0.318142	-2.217625
C	-0.731156	-0.119922	-1.264851
H	-1.236740	-0.318143	-2.217632
C	-1.430623	-0.078158	-0.103680
H	-2.518667	-0.220738	-0.103311
C	-0.724201	0.260273	1.198691
H	-0.721606	1.365381	1.321730
H	-1.276600	-0.152333	2.062151
C	0.724193	-0.260273	1.198696
H	0.721598	-1.365381	1.321735
H	1.276587	0.152333	2.062158

Table 5: R3 optimized geometries at B3LYP/6-31G* and MP2/aug-cc-pVDZ level of theory

(a) R and P, MP2

C	-0.25452	1.50213	-0.08789
C	0.25452	-1.50213	-0.08789
C	0.25452	0.7308	1.10044
C	-0.53006	-2.06965	-1.02957
C	0.53006	2.06965	-1.02957
C	-0.25452	-0.7308	1.10044
H	-0.07904	1.21977	2.03351
H	-0.10155	-2.6105	-1.87694
H	1.359	0.73899	1.10346
H	-1.62099	-2.00237	-0.97052
H	1.62099	2.00237	-0.97052
H	-1.359	-0.73899	1.10346
H	0.10155	2.6105	-1.87694
H	0.07904	-1.21977	2.03351
H	-1.34542	1.58492	-0.18742
H	1.34542	-1.58492	-0.18742

(b) R and P, DFT/B3LYP

C	-0.25184	1.54322	-0.10493
C	0.25184	-1.54322	-0.10493
C	0.25184	0.73198	1.05765
C	-0.513	-2.20013	-0.97705
C	0.513	2.20013	-0.97705
C	-0.25184	-0.73198	1.05765
H	-0.07625	1.2006	1.99765
H	-0.0865	-2.77542	-1.79452
H	1.34985	0.74498	1.0695
H	-1.59913	-2.18861	-0.90696
H	1.59913	2.18861	-0.90696
H	-1.34985	-0.74498	1.0695
H	0.0865	2.77542	-1.79452
H	0.07625	-1.2006	1.99765
H	-1.33705	1.58054	-0.21967
H	1.33705	-1.58054	-0.21967

(c) R and P, CCSD/jun-cc-pVDZ

C	-0.239559	1.532230	-0.104424
C	0.239559	-1.532230	-0.104424
C	0.260201	0.728091	1.074583
C	-0.549475	-2.159706	-1.000217
C	0.549475	2.159706	-1.000217
C	-0.260201	-0.728091	1.074583
H	-0.067580	1.216240	2.014202
H	-0.125671	-2.730822	-1.834575
H	1.367817	0.729851	1.082336
H	-1.644122	-2.118727	-0.924977
H	1.644122	2.118727	-0.924977
H	-1.367817	-0.729851	1.082336
H	0.125671	2.730822	-1.834575
H	0.067580	-1.216240	2.014202
H	-1.332957	1.593605	-0.221844
H	1.332957	-1.593605	-0.221844

(d) TS, MP2

C	1.412634	0.000454	-0.270789
C	-1.412634	-0.000454	0.270789
C	0.876961	-1.223498	0.249260
C	-0.876961	1.223498	-0.249260
C	0.876177	1.224058	0.249269
C	-0.876177	-1.224058	-0.249269
H	1.269720	-2.155789	-0.179442
H	-1.269720	2.155789	0.179442
H	0.783441	-1.282520	1.341538
H	-0.783441	1.282520	-1.341538
H	0.782618	1.283011	1.341548
H	-0.782618	-1.283011	-1.341548
H	1.268338	2.156604	-0.179425
H	-1.268338	-2.156604	0.179425
H	1.771384	0.000573	-1.309023
H	-1.771384	-0.000573	1.309023

(e) TS, DFT/B3LYP

C	1.431739	0.000460	-0.260245
C	-1.431739	-0.000460	0.260245
C	0.950420	-1.218276	0.253966
C	-0.950420	1.218276	-0.253966
C	0.949639	1.218884	0.253976
C	-0.949639	-1.218884	-0.253976
H	1.312013	-2.144839	-0.191698
H	-1.312013	2.144839	0.191698
H	0.814639	-1.299807	1.330983
H	-0.814639	1.299807	-1.330983
H	0.813806	1.300320	1.330993
H	-0.813806	-1.300320	-1.330993
H	1.310638	2.145682	-0.191681
H	-1.310638	-2.145682	0.191681
H	1.823869	0.000590	-1.277826
H	-1.823869	-0.000590	1.277826

(f) TS, CCSD/jun-cc-pVDZ

C	1.419737	0.000456	-0.266364
C	-1.419737	-0.000456	0.266364
C	0.913744	-1.224745	0.254008
C	-0.913744	1.224745	-0.254008
C	0.912959	1.225328	0.254018
C	-0.912959	-1.225328	-0.254018
H	1.289837	-2.158408	-0.190346
H	-1.289837	2.158408	0.190346
H	0.798278	-1.297439	1.344640
H	-0.798278	1.297439	-1.344640
H	0.797446	1.297940	1.344650
H	-0.797446	-1.297940	-1.344650
H	1.288453	2.159236	-0.190329
H	-1.288453	-2.159236	0.190329
H	1.800575	0.000582	-1.298542
H	-1.800575	-0.000582	1.298542

2 Scalability Tests

The here reported tests with the Gaussian16 software have been run using the following machines:

CINECA GALILEO NeXtScale cluster CentOS 7.4: 1022 compute nodes with 2×18 core Intel Xeon E5-2697 v4 (Broadwell) at 2.30 GHz 128 GB RAM.[2]

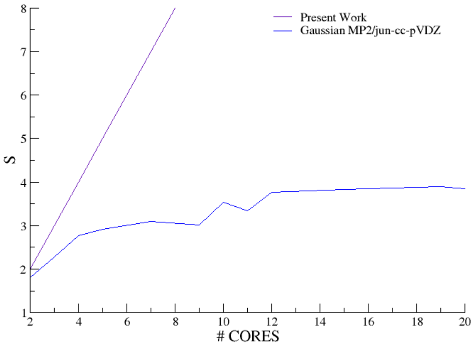

(a) MP2 Speedup plot.

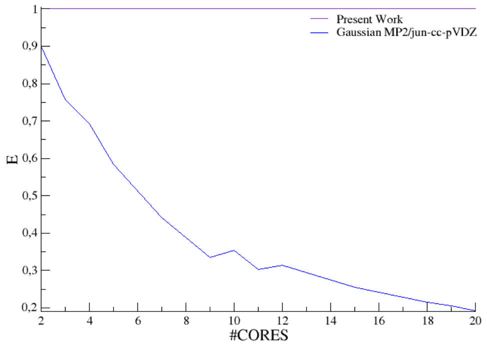

(b) MP2 Efficiency plot.

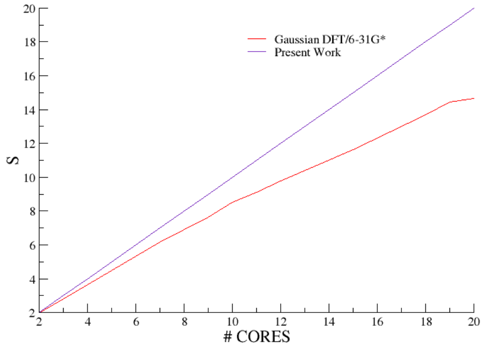

(c) DFT Speedup plot.

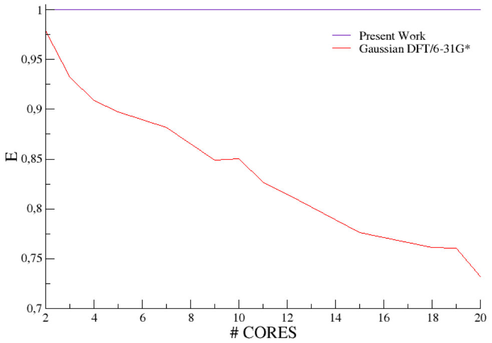

(d) DFT Efficiency plot.

Figure 1: Speedup and Efficiency plots for the anharmonic constants calculation using Gaussian16. Calculations are done on the 10 atoms **cyclobutene** molecule using the MP2 post-HF method with the jun-cc-pVDZ and the DFT/B3LYP with the 6-31G* Basis Set (BS).

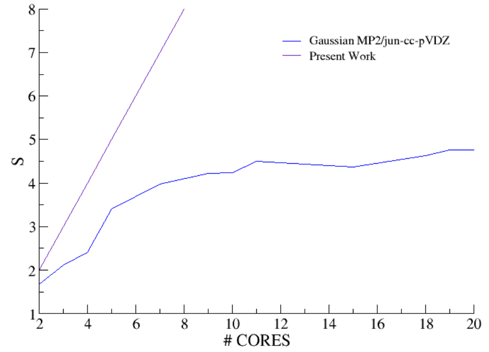

(a) MP2 Speedup plot.

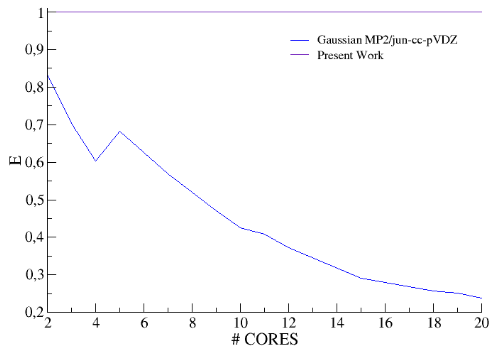

(b) MP2 Efficiency plot.

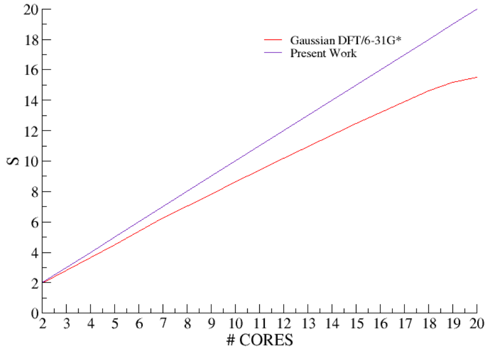

(c) DFT Speedup plot.

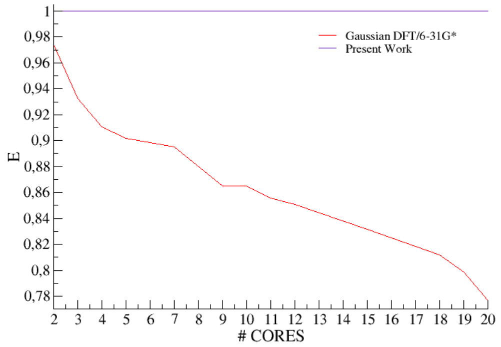

(d) DFT Efficiency plot.

Figure 2: Speedup and Efficiency plots for the anharmonic constants calculation using Gaussian16. Calculations are done on the 14 atoms **cis-1,3,5-hexatriene** molecule using the MP2 post-HF method with the jun-cc-pVDZ and the DFT/B3LYP with the 6-31G* Basis Set (BS).

3 Accuracy Tests

We report here the results from the direct comparison of our anharmonic constants with the ones obtained using Gaussian16. The definitions of the difference $\Delta\chi$ and the percentage difference $\%_{diff}$ are given in the article in eq. 30.

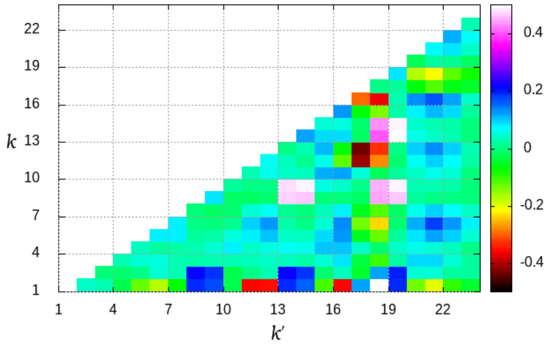

(a) DFT difference $\Delta\chi$

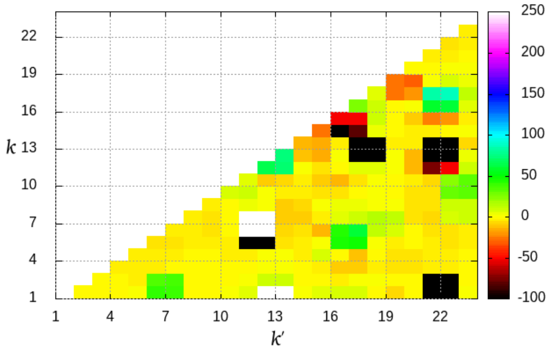

(b) DFT percentage difference $\%_{diff}$

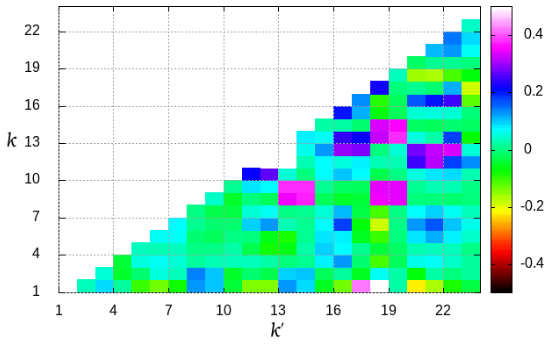

(c) MP2 difference $\Delta\chi$

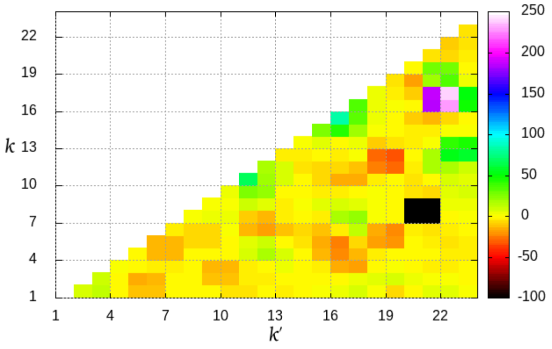

(d) MP2 percentage difference $\%_{diff}$

Figure 3: Comparison between anharmonic constants calculated with Gaussian16 and our program for the **cyclobutene** molecule. The horizontal and vertical axes indicate the normal mode indexes for the $\chi_{k,k'}$ and the colour gradient indicates the magnitude of the difference between the results. Anharmonic constants are calculated using the DFT/B3LYP functional with the 6-31G* Basis Set (BS) and the MP2 post-HF method with the aug-cc-pVDZ BS.

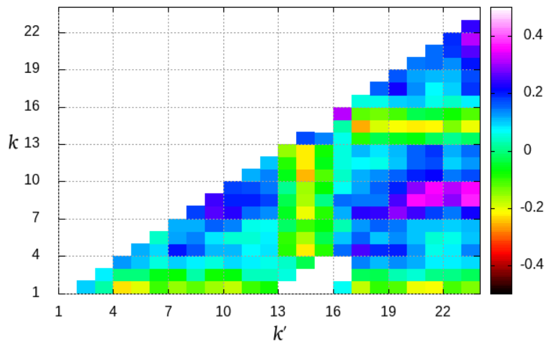

(a) DFT difference $\Delta\chi$

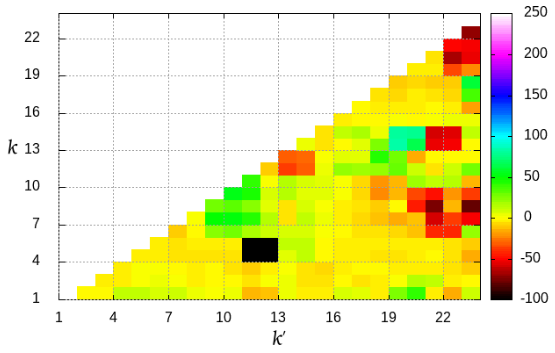

(b) DFT percentage difference $\%_{diff}$

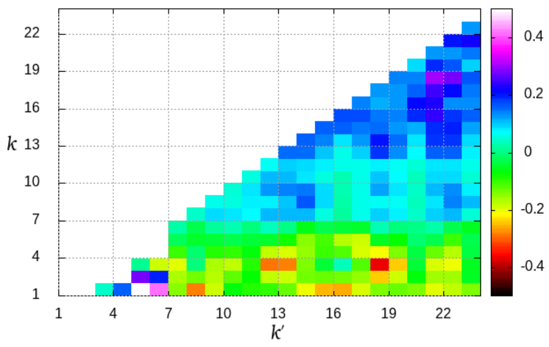

(c) MP2 difference $\Delta\chi$

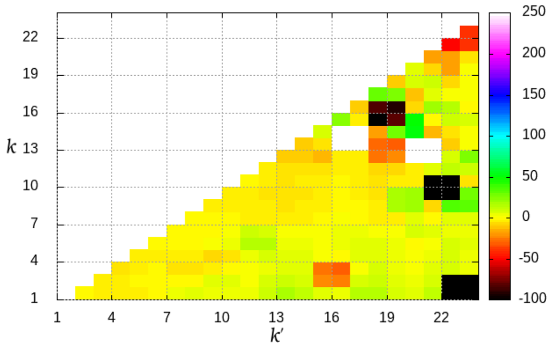

(d) MP2 percentage difference $\%_{diff}$

Figure 4: Comparison between anharmonic constants calculated with Gaussian16 and our program for the **cyclobutene** opening reaction transition state. The horizontal and vertical axes indicate the normal mode indexes for the $\chi_{k,k'}$ and the colour gradient indicates the magnitude of the difference between the results. Anharmonic constants are calculated using the DFT/B3LYP functional with the 6-31G* Basis Set (BS) and the MP2 post-HF method with the aug-cc-pVDZ BS.

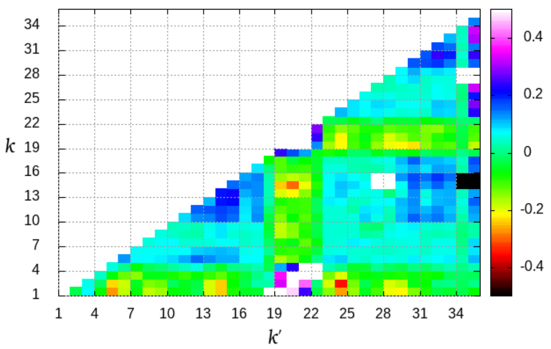

(a) DFT difference $\Delta\chi$

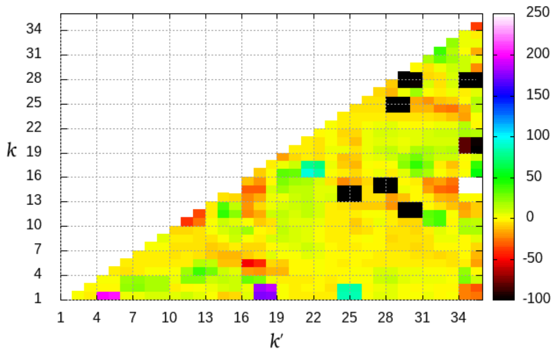

(b) DFT percentage difference $\%_{diff}$

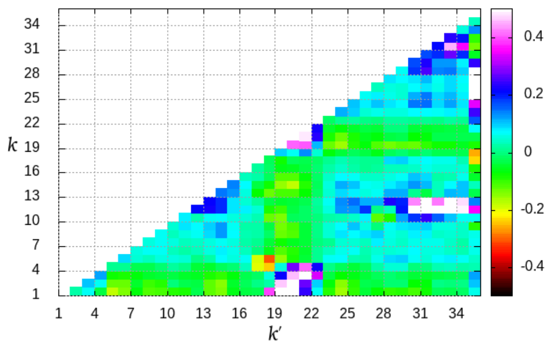

(c) MP2 difference $\Delta\chi$

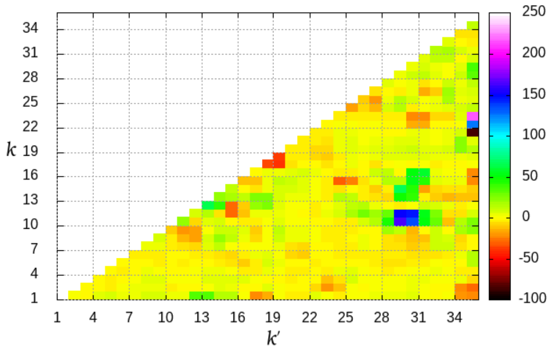

(d) MP2 percentage difference $\%_{diff}$

Figure 5: Comparison between anharmonic constants calculated with the Gaussian16 software and our program for the TS molecule of the **hexatriene** ring closing electrocyclic reaction. The horizontal and vertical axes indicate the normal mode indexes for the $\chi_{k,k'}$ and the colour gradient indicates the magnitude of the difference between the results. Anharmonic constants are calculated using the DFT/B3LYP functional with the 6-31G* Basis Set (BS) and the MP2 post-HF method with the aug-cc-pVDZ BS.

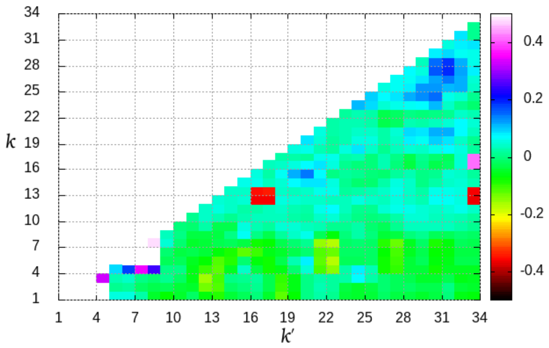

(a) DFT difference $\Delta\chi$

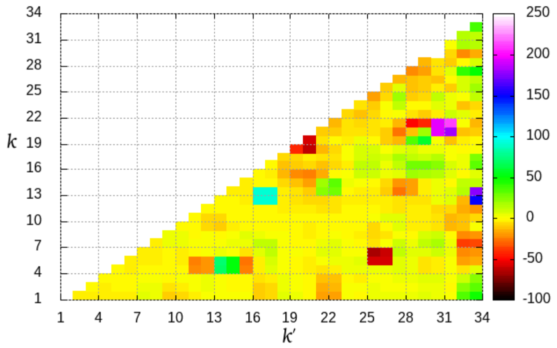

(b) DFT percentage difference $\%_{diff}$

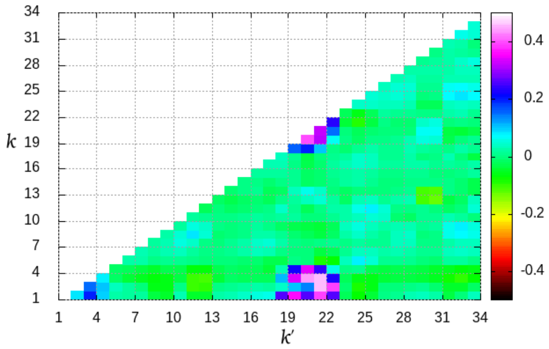

(c) MP2 difference $\Delta\chi$

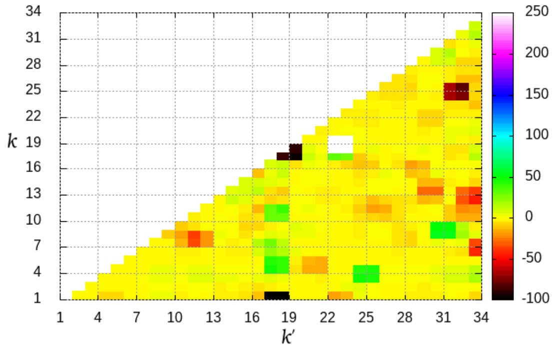

(d) MP2 percentage difference $\%_{diff}$

Figure 6: Comparison between anharmonic constants calculated with Gaussian and our program for the C_2 **hexatriene** molecule. The horizontal and vertical axes indicate the normal mode indexes for the $\chi_{k,k'}$ and the colour gradient indicates the magnitude of the difference between the results. Anharmonic constants are calculated using the DFT/B3LYP functional with the 6-31G* Basis Set (BS) and the MP2 post-HF method with the aug-cc-pVDZ BS.

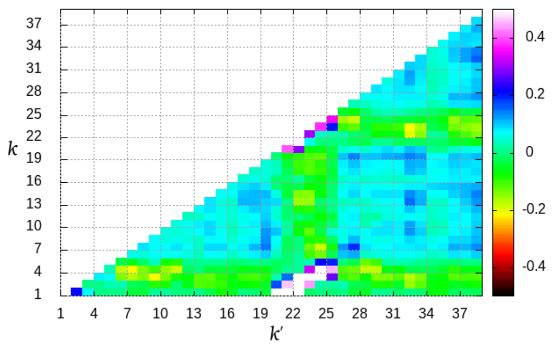

(a) DFT difference $\Delta\chi$

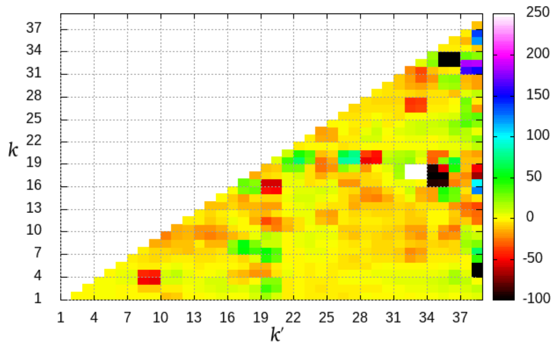

(b) DFT percentage difference $\%_{diff}$

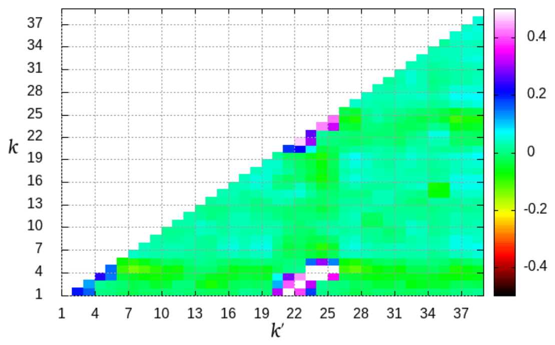

(c) MP2 difference $\Delta\chi$

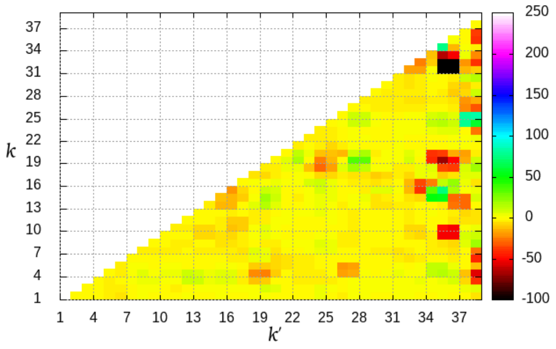

(d) MP2 percentage difference $\%_{diff}$

Figure 7: Comparison between anharmonic constants calculated with Gaussian16 and our program for the **1,5-hexadiene** molecule. The horizontal and vertical axes indicate the normal mode indexes for the $\chi_{k,k'}$ and the colour gradient indicates the magnitude of the difference between the results. Anharmonic constants are calculated using the DFT/B3LYP functional with the 6-31G* Basis Set (BS) and the MP2 post-HF method with the aug-cc-pVDZ BS.

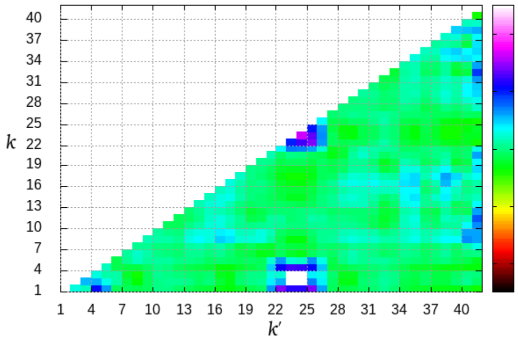

(a) DFT difference $\Delta\chi$

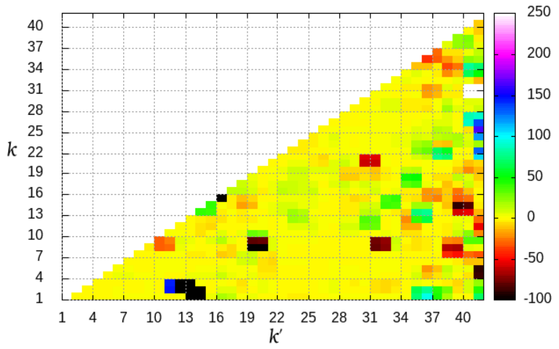

(b) DFT percentage difference $\%_{diff}$

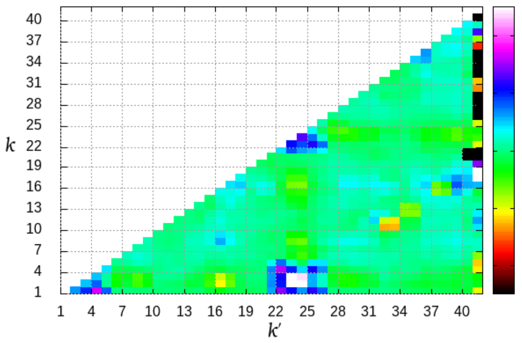

(c) MP2 difference $\Delta\chi$

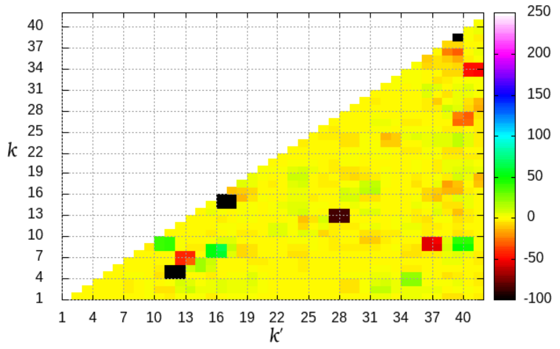

(d) MP2 percentage difference $\%_{diff}$

Figure 8: Comparison between anharmonic constants calculated with Gaussian16 and our program for the TS molecule of the **1,5-hexadiene** Cope rearrangement. The horizontal and vertical axes indicate the normal mode indexes for the $\chi_{k,k'}$ and the colour gradient indicates the magnitude of the difference between the results. Anharmonic constants are calculated using the DFT/B3LYP functional with the 6-31G* Basis Set (BS) and the MP2 post-HF method with the aug-cc-pVDZ BS.

The impact on the SCTST reaction rate constants of small variations between the anharmonic constants calculated with our program and the Gaussian software is shown in the following results for the three reactions studied in our paper.

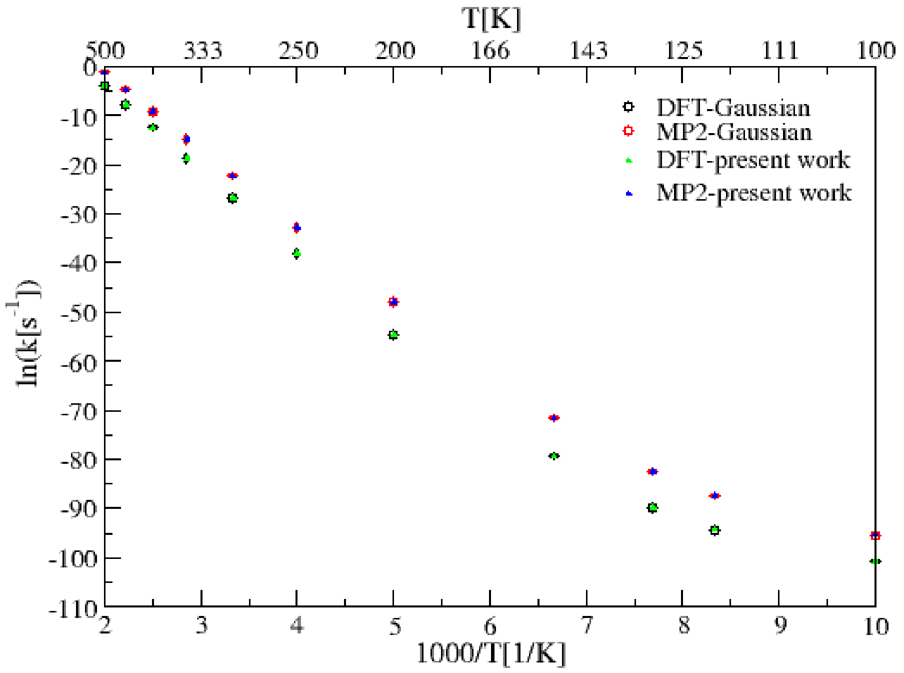

Figure 9: SCTST Reaction Rate Constants at different temperatures for the R1 reaction. The anharmonic constants matrix was calculated using our program and the Gaussian16 software starting from the same geometry and ab initio calculation options. DFT calculations have been carried out using the B3LYP functional with the 6-31G* basis set while in the MP2 calculations we used the aug-cc-pVDZ one.

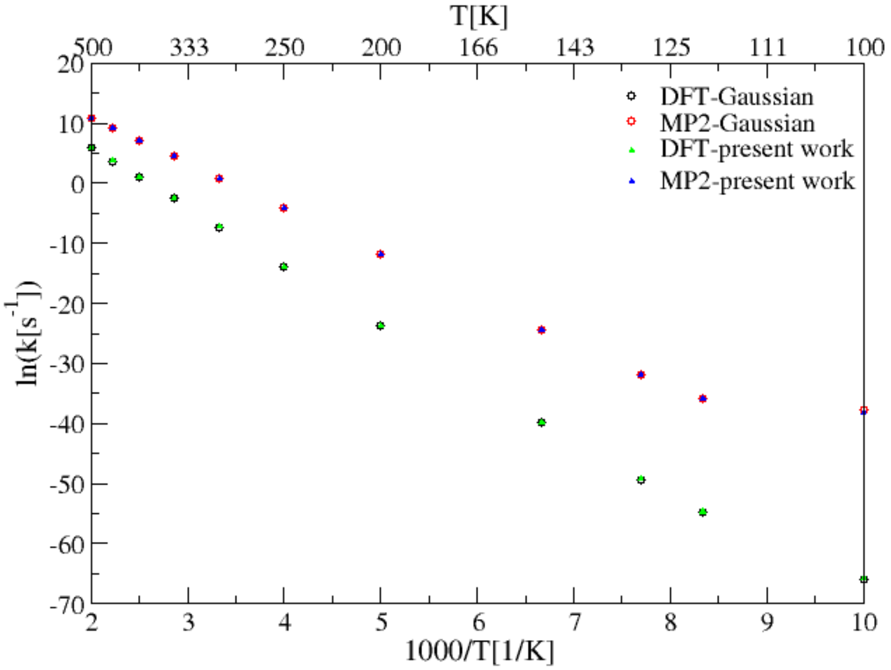

Figure 10: SCTST Reaction Rate Constants at different temperatures for the R2 reaction. The anharmonic constants matrix was calculated using our program and the Gaussian16 software starting from the same geometry and ab initio calculation options. DFT calculations have been carried out using the B3LYP functional with the 6-31G* basis set, while in the MP2 calculations we used the aug-cc-pVDZ one.

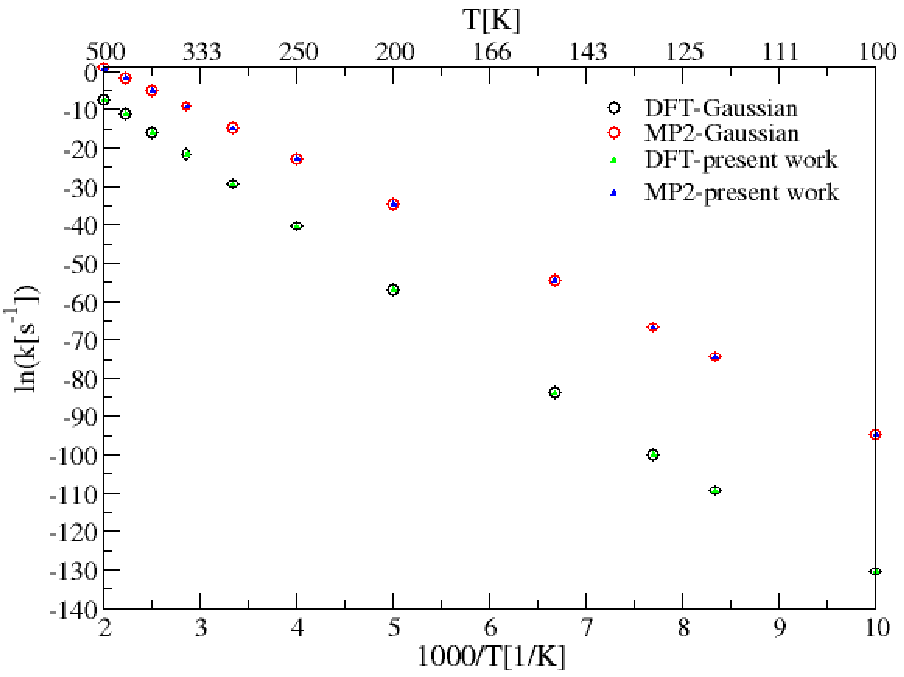

Figure 11: SCTST Reaction Rate Constants at different temperatures for the R3 reaction. The anharmonic constants matrix was calculated using our program and the Gaussian16 software starting from the same geometry and ab initio calculation options. DFT calculations have been carried out using the B3LYP functional with the 6-31G* basis set, while in the MP2 calculations we used the aug-cc-pVDZ basis set.

4 Energetic of the Reactions

The barriers here reported are the classical barriers corrected for the harmonic Zero Point Energy (ZPE): $E^{R}_{harmZPE} = \frac{1}{2}\sum_{k=1}^{N}\omega_k$, $E^{TS}_{harmZPE} = \frac{1}{2}\sum_{k=1}^{N-1}\omega_k$ and corrected for the anharmonic ZPE: $E^{R}_{anharmZPE} = \frac{1}{2}\sum_{k=1}^{N}\omega_k + \frac{1}{4}\sum_{k,k'=1}^{N}\chi_{k,k'} + \mathcal{G}_0^R$ and $E^{TS}_{anharmZPE} = \frac{1}{2}\sum_{k=1}^{N-1}\omega_k + \frac{1}{4}\sum_{k,k'=1}^{N-1}\chi_{k,k'} + \mathcal{G}_0^{TS}$ where N is the number of vibrational degrees of freedom, ω_k are the harmonic vibrational frequencies, $\mathcal{G}_0^R$ and $\mathcal{G}_0^{TS}$ are the constant terms coming from the VPT2 perturbation expansion and R and TS refer to the formula for the reactant and the transition state.

Table 6: Harmonic and anharmonic ZPE corrected forward (E_f) and backward (E_b) reaction barriers and TS reactive mode harmonic frequencies ($\tilde{\omega}$) for the R1reaction.

Method/BS	$E_f(ZPE_{harm}; ZPE_{anhar})$ /[kJmol^{-1}]	E_b /[kJmol^{-1}]	$\tilde{\omega}$ /[cm−1]
B3LYP/6-31G*	141.769; 141.592	195.143	742.429
MP2/aug-cc-pVDZ	130.561; 130.248	175.961	713.852
CCSD(T)/aug-cc-pVDZ	132.407; 131.551	185.975	730.328

Table 7: Harmonic and anharmonic ZPE corrected forward (E_f) and backward (E_b) reaction barriers and TS reactive mode harmonic frequencies ($\tilde{\omega}$) for the R2 reaction.

Method/BS	$E_f(ZPE_{harm}; ZPE_{anhar})$ /[kJmol^{-1}]	E_b /[kJmol^{-1}]	$\tilde{\omega}$ /[cm−1]
B3LYP/6-31G*	86.692; 86.585	179.028	571.875
MP2/aug-cc-pVDZ	67.549; 68.182	170.841	443.303
CCSD/jun-cc-pVDZ	116.063; 116.087	208.533	746.523
CCSD/aug-cc-pVDZ	110.570		737.491

Table 8: Harmonic and anharmonic ZPE corrected forward (E_f) and backward (E_b) reaction barriers and TS reactive mode harmonic frequencies ($\tilde{\omega}$) for the R3 reaction.

Method/BS	$E_f(ZPE_{harm}; ZPE_{anhar})$ /[kJmol^{-1}]	E_b /[kJmol^{-1}]	$\tilde{\omega}$ /[cm−1]
B3LYP/6-31G*	141.682; 141.451	140.031	569.246
MP2/aug-cc-pVDZ	102.317; 109.111	100.840	110.546
CCSD/jun-cc-pVDZ	174.181; 173.940	172.511	696.535

5 Hindered Rotations Treatment

For the hindered rotational modes (HRM) treatment, involved in the low frequency modes of the R2 and R3 reactants, we used the Pitzer and Gewin (PG) coefficients for the correction of the harmonic oscillator partition function.

The overall correction values for the total harmonic partition functions associated with the HRM are listed in Tables (9) and (10).

Table 9: Overall PG correction coefficients for the HRM in the R2 reaction at the MP2/aug-cc-pVDZ, B3LYP/6-31G* and CCSD/jun-cc-pVDZ level of theory.

(a) MP2 PG coefficients.

$\omega_1/[\mathrm{cm}^{-1}]$	100.213
$\omega_2/[\mathrm{cm}^{-1}]$	103.936
Multiplicity	1.000
T/[K]	Overall PG coefficient
100	1.018
120	1.022
130	1.024
150	1.028
200	1.039
250	1.052
300	1.065
350	1.080
400	1.095
450	1.110
500	1.125

(b) DFT PG coefficients.

$\omega_1/[\mathrm{cm}^{-1}]$	55.059
$\omega_2/[\mathrm{cm}^{-1}]$	93.797
Multiplicity	1.000
T/[K]	Overall PG coefficient
100	1.024
120	1.029
130	1.032
150	1.038
200	1.054
250	1.071
300	1.090
350	1.110
400	1.130
450	1.148
500	1.165

(c) CCSD PG coefficients.

$\omega_1/[\mathrm{cm}^{-1}]$	93.754
$\omega_2/[\mathrm{cm}^{-1}]$	97.654
Multiplicity	1.000
T/[K]	Overall PG coefficient
100	1.022
120	1.027
130	1.029
150	1.034
200	1.048
250	1.064
300	1.081
350	1.098
400	1.116
450	1.134
500	1.151

Table 10: Overall PG correction coefficients for the HRM in the R3 reaction at the MP2/aug-cc-pVDZ, B3LYP/6-31G* and CCSD/jun-cc-pVDZ level of theory.

(a) MP2 PG coefficients.

$\omega_1/[\text{cm}^{-1}]$	51.106
$\omega_2/[\text{cm}^{-1}]$	90.513
$\omega_3/[\text{cm}^{-1}]$	105.307
Multiplicity	2.000
T/[K]	Overall PG coefficient
100	2.211
120	2.268
130	2.298
150	2.359
200	2.511
250	2.647
300	2.758
350	2.845
400	2.908
450	2.952
500	2.977

(b) DFT PG coefficients.

$\omega_1/[\text{cm}^{-1}]$	65.675
$\omega_2/[\text{cm}^{-1}]$	101.710
$\omega_3/[\text{cm}^{-1}]$	108.148
Multiplicity	2.000
T/[K]	Overall PG coefficient
100.000	2.150
120.000	2.188
130.000	2.208
150.000	2.251
200.000	2.372
250.000	2.501
300.000	2.629
350.000	2.748
400.000	2.851
450.000	2.939
500.000	3.009

(c) CCSD PG coefficients.

$\omega_1/[\text{cm}^{-1}]$	65.916
$\omega_2/[\text{cm}^{-1}]$	99.219
$\omega_3/[\text{cm}^{-1}]$	114.051
Multiplicity	2.000
T/[K]	Overall PG coefficient
100.000	2.144
120.000	2.179
130.000	2.199
150.000	2.239
200.000	2.354
250.000	2.478
300.000	2.601
350.000	2.715
400.000	2.816
450.000	2.901
500.000	2.971

6 Kinetic Rate Constants

For the computation of the reactants and the TS Density of State (DOS) with the Paradensum and Parsctst program[3–5] we used the following parameters:

Number of Walkers	1
Window Overlap	70 %
Flatness	95 %
Windows Balance	constant
Energy Grain / [cm^{-1}]	5
Upper Energy Limit	30000
Number of Elements in the Double Array	2500

The absolute values of the rate constants for the three reactions at different temperatures and ab initio levels of theory are here shown in the reported tables[1].

Table 11: TST and SCTST reaction rate constants at the MP2/aug-cc-pVDZ, CCSD(T)/aug-cc-pVDZ and B3LYP/6-31G* level of theory for the R1 reaction.

T/[K]	$k_{TST}^{DFT}(T)/[s^{-1}]$	$k_{SCTST}^{DFT}(T)/[s^{-1}]$	$k_{TST}^{MP2}(T)/[s^{-1}]$	$k_{SCTST}^{MP2}(T)/[s^{-1}]$
100	2.11675E-062	2.13928E-044	1.49633E-056	6.29877E-042
120	5.53349E-050	1.45661E-041	4.11002E-045	1.524785E-038
130	3.32983E-045	1.19574E-039	1.03871E-040	1.94188E-036
150	1.49548E-037	3.65046E-035	1.16246E-033	9.65712E-032
200	4.26616E-025	2.28611E-024	3.44999E-022	1.71203E-021
250	1.32929E-017	3.43512E-017	2.75416E-015	7.22676E-015
300	1.36253E-012	2.57491E-012	1.13615E-010	2.24878E-010
350	5.30245E-009	8.44840E-009	2.30435E-007	3.91287E-007
400	2.66162E-006	3.81852E-006	7.08679E-005	1.09741E-004
450	3.40065E-004	4.55119E-004	6.17995E-003	9.02378E-003
500	1.66349E-002	2.12066E-002	0.22255	0.31260

T/[K]	$k_{TST}^{CCSD(T)}(T)/[s^{-1}]$	$k_{SCTST}^{CCSD(T)}(T)/[s^{-1}]$
100	1.63964E-057	8.54513E-039
120	6.53249E-046	4.42661E-037
130	1.90549E-041	1.29039E-035
150	2.68434E-034	9.86615E-032
200	1.16160E-022	9.21670E-022
250	1.16707E-015	4.25952E-015
300	5.62845E-011	1.45315E-010
350	1.27921E-007	2.72615E-007
400	4.29217E-005	8.13169E-005
450	4.01068E-003	7.03969E-003
500	0.15279	0.25482

[1]Values here reported refer to kinetic rate constants calculated using the anharmonic couplings obtained with our program.

Table 12: TST and SCTST reaction rate constants at the MP2/aug-cc-pVDZ, B3LYP/6-31G* and CCSD/jun-cc-pVDZ level of theory for the R2 reaction.

T/[K]	$k_{TST}^{DFT}(T)$/[s^{-1}]	$k_{SCTST}^{DFT}(T)$/[s^{-1}]	$k_{TST}^{MP2}(T)$/[s^{-1}]	$k_{SCTST}^{MP2}(T)$/[s^{-1}]
100	4.03881E-034	1.13132E-029	9.48905E-024	5.13281E-018
120	1.40522E-026	8.74494E-025	7.74509E-018	4.17104E-017
130	1.10653E-023	2.06538E-022	1.44387E-015	2.33636E-015
150	4.68877E-019	2.78935E-018	6.11954E-012	5.42185E-012
200	1.45733E-011	3.29232E-011	4.55622E-006	2.38607E-006
250	4.31711E-007	6.85389E-007	1.44938E-002	6.09146E-003
300	3.99407E-004	5.28686E-004	3.05101	1.12436
350	5.14018E-002	6.09214E-002	136.78973	45.89433
400	1.93873	2.13278	2345.40624	730.52197
450	32.41599	33.78148	21240.54872	6212.91933
500	307.11192	307.00269	123225.99859	34104.14281

T/[K]	$k_{TST}^{CCSD}(T)$/[s^{-1}]	$k_{SCTST}^{CCSD}(T)$/[s^{-1}]
100	3.23214E-049	1.33064E-040
120	4.29893E-039	9.79458E-035
130	3.33972E-035	3.96741E-032
150	5.51741E-029	2.15141E-027
200	6.62577E-019	1.98934E-018
250	7.01376E-013	1.02646E-012
300	7.05351E-009	7.32838E-009
350	4.99767E-006	4.23627E-006
400	6.77155E-004	5.00719E-004
450	3.05601E-002	2.04616E-002
500	0.64053	0.39729

Table 13: TST and SCTST reaction rate constants at the MP2/aug-cc-pVDZ, B3LYP/6-31G* and CCSD/jun-cc-pVDZ level of theory for the R3 reaction.

T/[K]	$k_{TST}^{DFT}(T)/[s^{-1}]$	$k_{SCTST}^{DFT}(T)/[s^{-1}]$	$k_{TST}^{MP2}(T)/[s^{-1}]$	$k_{SCTST}^{MP2}(T)/[s^{-1}]$
100	5.14108E-063	4.40637E-058	1.38839E-042	3.61820E-046
120	1.02706E-050	7.23459E-049	9.94086E-034	1.00828E-036
130	5.46408E-046	1.09358E-044	2.49353E-030	4.25883E-033
150	1.95583E-038	1.26557E-037	6.71034E-025	2.63201E-027
200	3.46576E-026	8.89942E-026	4.16946E-016	6.25655E-018
250	7.38090E-019	1.37385E-018	7.48352E-011	2.48835E-012
300	5.53659E-014	8.84309E-014	2.33016E-007	1.30769E-008
350	1.66324E-010	2.44717E-010	7.22687E-005	5.86216E-006
400	6.73489E-008	9.46018E-008	5.33657E-003	5.68098E-004
450	7.18979E-006	9.83407E-006	0.15179	1.98863E-002
500	3.02751E-004	4.07996E-004	2.21997	0.34226

T/[K]	$k_{TST}^{CCSD}(T)/[s^{-1}]$	$k_{SCTST}^{CCSD}(T)/[s^{-1}]$
100	5.38555E-080	1.49492E-045
120	7.28489E-065	2.14694E-045
130	4.75194E-059	2.80869E-045
150	9.39617E-050	8.32189E-045
200	1.12907E-034	5.72588E-034
250	1.20154E-025	3.20860E-025
300	1.22244E-019	2.50631E-019
350	2.36386E-015	4.23288E-015
400	3.86298E-012	6.41795E-012
450	1.22057E-009	1.94476E-009
500	1.22293E-007	1.90647E-007

The values of the % difference between the SCTST and the TST rate constants are reported in the following tables.

The definition of the % difference can be found in the article in eq. 33.

Table 14: % difference at the MP2/aug-cc-pVDZ, CCSD(T)/aug-cc-pVDZ, B3LYP/6-31G* and CCSD/jun-cc-pVDZ level of theory for the R1, R2 and R3 reactions.

(a) R1 % difference.

T/[K]	% Diff DFT	% Diff MP2	% Diff CCSD(T)
100	100.000	99.999	100.000
120	99.999	99.999	99.999
130	99.999	99.994	99.999
150	99.590	98.796	99.728
200	81.339	79.848	87.397
250	61.303	61.889	72.601
300	47.084	49.477	61.267
350	37.237	41.108	53.076
400	30.297	35.422	47.217
450	25.279	31.515	43.028
500	21.558	28.807	40.037

(b) R2 % difference.

T/[K]	% Diff DFT	% Diff MP2	% Diff CCSD
100	99.996	99.999	99.999
120	98.393	81.431	99.996
130	94.642	38.200	99.916
150	83.190	-12.868	97.435
200	55.735	-90.951	66.694
250	37.012	-137.936	31.670
300	24.453	-171.357	3.751
350	15.626	-198.054	-17.973
400	9.098	-221.059	-35.236
450	4.042	-241.877	-49.353
500	-0.036	-261.323	-61.222

(c) R3 % difference.

T/[K]	% Diff DFT	% Diff MP2	% Diff CCSD
100	99.998	-383623.007	100.000
120	98.580	-98492.313	100.000
130	95.003	-58449.588	99.999
150	84.546	-25395.067	99.998
200	61.056	-6564.150	80.281
250	46.276	-2907.423	62.552
300	37.391	-1681.889	51.225
350	32.034	-1132.799	44.155
400	28.808	-839.375	39.809
450	26.889	-663.272	37.238
500	25.796	-548.612	35.854

References

(1) Frisch, M. J. et al. Gaussian16 Revision C.01. **2016**, Gaussian Inc. Wallingford CT.

(2) GALILEO IBM NeXtScale cluster. https://www.hpc.cineca.it/hardware/galileo.

(3) Aieta, C.; Gabas, F.; Ceotto, M. An efficient computational approach for the calculation of the vibrational density of states. *The Journal of Physical Chemistry A* **2016**, *120*, 4853–4862.

(4) Aieta, C.; Gabas, F.; Ceotto, M. Parallel Implementation of Semiclassical Transition State Theory. *Journal of chemical theory and computation* **2019**, *15*, 2142–2153.

(5) Barker, J.; Nguyen, T.; Stanton, J.; Aieta, C.; Ceotto, M.; F. Gabas, T. K.; Li, C.; Lohr, L.; Maranzana, A.; Ortiz, N.; Preses, J.; Simmie, J.; Sonk, J.; ; Stimac, P. MultiWell-2020 Software Suite; J. R. Barker, University of Michigan, Ann Arbor, Michigan, USA, 2020. http://clasp-research.engin.umich.edu/multiwell/.
